# A replicator-specific binding protein essential for site-specific initiation of DNA replication in mammalian cells

**DOI:** 10.1038/ncomms11748

**Published:** 2016-06-08

**Authors:** Ya Zhang, Liang Huang, Haiqing Fu, Owen K. Smith, Chii Mei Lin, Koichi Utani, Mishal Rao, William C. Reinhold, Christophe E. Redon, Michael Ryan, RyangGuk Kim, Yang You, Harlington Hanna, Yves Boisclair, Qiaoming Long, Mirit I. Aladjem

**Affiliations:** 1 Developmental Therapeutics Branch, Center for Cancer Research, National Cancer Institute, National Institutes of Health, Bethesda, Maryland 20892, USA; 2 In Silico Solutions, Fairfax, Virginia 22033, USA; 3 Department of Animal Science, Cornell University, Ithaca, New York 14853-4801, USA

## Abstract

Mammalian chromosome replication starts from distinct sites; however, the principles governing initiation site selection are unclear because proteins essential for DNA replication do not exhibit sequence-specific DNA binding. Here we identify a replication-initiation determinant (RepID) protein that binds a subset of replication-initiation sites. A large fraction of RepID-binding sites share a common G-rich motif and exhibit elevated replication initiation. RepID is required for initiation of DNA replication from RepID-bound replication origins, including the origin at the human beta-globin *(HBB)* locus. At *HBB*, RepID is involved in an interaction between the replication origin (Rep-P) and the locus control region. RepID-depleted murine embryonic fibroblasts exhibit abnormal replication fork progression and fewer replication-initiation events. These observations are consistent with a model, suggesting that RepID facilitates replication initiation at a distinct group of human replication origins.

## Abstract (web summary)

Origins of mammalian DNA replication are poorly characterised because they lack an Identifiable consensus sequence. Here the authors identify RepID, a protein that binds to a subset of G-rich replication origins and facilitates initiation from those origins.

## Graphical abstract

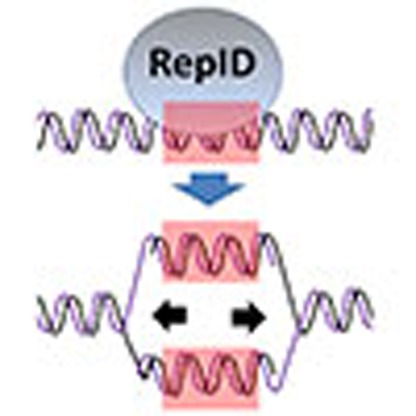

All eukaryotic proliferating cells duplicate their entire genome with high fidelity during the S phase of the cell cycle. This duplication requires precise coordination between DNA replication, cell cycle progression, chromatin remodelling and transcription. In eukaryotes, DNA replication initiates from distinct sites within the genome (replication origins), which can vary depending on the cell type and developmental stage^1^,^2^,^3^,^4^,^5^,^6^,^7^. To initiate DNA replication, a licensing step is required^8^. Following mitosis, chromatin-bound origin recognition complex (ORC) proteins recruit the licensing factors CDC6 and CDT1 to facilitate the loading of minichromosome maintenance helicases, and the formation of a pre-replication complex^9^,^10^,^11^. Specific ORC-binding determines replication origin activity in yeast; however, in metazoans, proteins that are essential for replication do not bind to specific DNA sequences^12^,^13^. Pre-replication complexes modify nucleosome-positioning in both *Drosophila*^14^ and vertebrates^15^, and the ORC1 member of the pre-replication complex binds preferentially to open chromatin^16^. However, these associations are not sufficient to determine the locations of all replication-initiation sites, and it is therefore unclear how nonspecific binding of pre-replication complexes might determine where and when metazoan replication initiates. In addition, the factors that coordinate replication with other cellular processes (that is, cell cycle progression) require further characterization.

There are indications that additional proteins, which are not members of the pre-replication complex, participate in specific DNA–protein interactions occurring at replication-initiation sites. Of particular interest are proteins that bind replicator sequences. Replicators are defined as DNA sequences that contain genetic information, which allows them to function as replication origins^17^. To date, only a few replicators have been identified in the metazoan genome. Although replication-initiation sites share common characteristics^18^, no consensus sequences have been identified among known replicators^3^,^8^,^10^,^18^,^19^. Only a small subset of the potential replication origins initiate DNA replication in each cell cycle^1^,^3^,^10^, and the choice of origins that initiate each cell cycle is yet unclear. Proteins that interact with particular replicators may thus play a role in determining where and when replication starts. Examples include the interaction of the *c-MYC* replicator with a DNA-unwinding element-specific protein that recruits CDC45 (ref. 20), interactions of RecQ1 and RecQ4 helicases with pre-initiation complexes^20^,^21^, interactions of the histone H3 lysine 9 methyltransferase-associated ORCA protein with pre-replication complexes^22^ and the role of histone acetylase HBO1 in the activation of CDT1 (ref. 23). Although no sequence-specific DNA-binding proteins were yet shown to determine replicator-specific initiation in mammalian cells, these studies indicate that distinct proteins might interact with subsets of origins, and recruit the general replication machinery to those sites.

The diverse family of WD40-repeat-containing proteins (DDB1- and CUL4-associated factors (DCAFs)) includes Pleckstrin Homology domain-Interacting Protein (PHIP), also known as DCAF14, which associates with insulin receptor substrate (IRS)-1 and IRS-2 (ref. 24). DCAF14/PHIP has also been shown to stimulate cell proliferation and to inhibit apoptosis^25^,^26^, and it can serve as a marker for aggressive metastatic melanoma^27^. One member of the pre-replication complex, CDT1, is a known substrate for a DCAF, CDT2, which mediates its ubiquitination by Cullin 4 (CUL4) and Damage-specific DNA-Binding protein 1 (DDB1)^28^ and facilitates its degradation in a p97-dependent pathway^29^,^30^.

The human *beta-globin* locus (*HBB*) contains two intensely studied replicators residing in the replication-initiation regions (IRs)^31^,^32^,^33^,^34^,^35^. This IR is used in both erythroid and non-erythroid cells, but initiates DNA replication during early stages of the S phase in erythroid cells and later during the S phase in non-erythroid cells^11^,^36^,^37^,^38^,^39^,^40^. Each of the two replicators (Rep-P and RepI) within *HBB* IR can initiate DNA replication at both native and ectopic sites^31^,^34^. Each replicator contains an AT-rich sequence and an asymmetric purine, pyrimidine (AG) sequence, with both sequences required for replication initiation^34^,^35^. The *HBB* IR was used to drive replication of human artificial chromosomes^32^ and as a model replication origin in evolutionary, biochemical and functional studies^21^,^35^,^41^,^42^,^43^,^44^. The *HBB* IR, therefore, provides an excellent system to study replicator-binding proteins as well as an opportunity to study replication timing.

To better understand sequence-specific replication initiation, we used the *HBB* IR as a model to capture replicator-binding proteins. We were particularly interested in proteins that bind to the essential asymmetric purine:pyrimidine (AG) region of the Rep-P replicator. Here we identify a protein RepID (replication-initiation determinant), also known as PHIP or DCAF14, which binds AG and is required for the initiation of DNA replication from Rep-P. Mutations in the RepID-binding sites disable replication initiation from Rep-P and RepID deficiency affects cell growth and reduces the frequency of replication initiation events genome-wide. RepID-binding is not restricted to Rep-P, as this protein binds other replication initiation sites that share a common sequence motif. These observations suggest that RepID may play multiple roles during the DNA replication process, functioning at specific categories of replication origins. As a member of the DCAF family and an IRS-1/2 interacting protein, RepID may also serve as a link between DNA replication and metabolic signalling pathways.

## Results

### The Rep-P asymmetric region binds RepID

**Figure 1:**
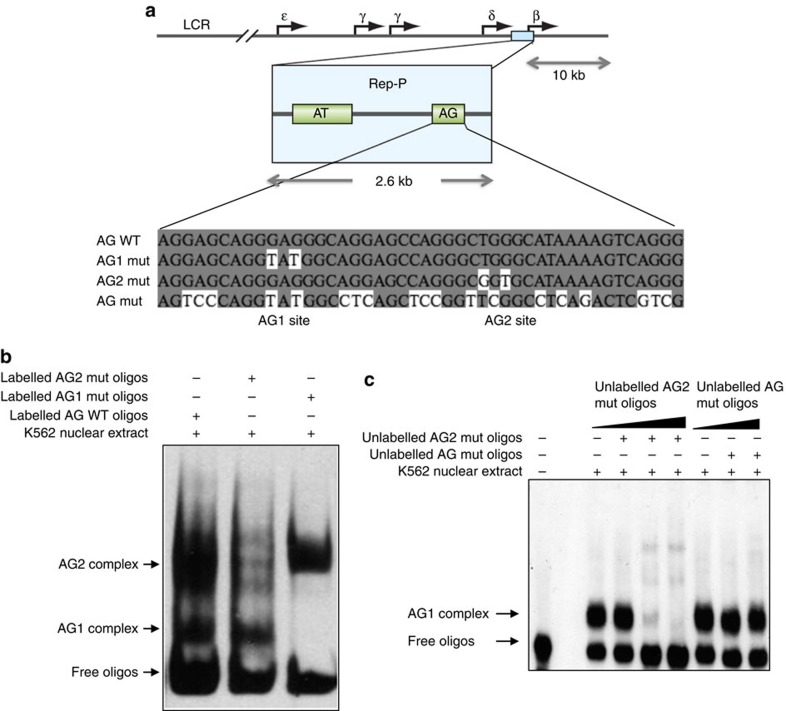
Two distinct DNA–protein interactions at replicator sequences. (**a**) Map of the *HBB* locus (top row), Rep-P (second row) and sequences of the intact (AG WT) and mutant (AG1, AG2 and AG mut) oligonucleotides used in this study. Only one strand is shown. The unshaded nucleotides indicate changes from the AG WT oligo. (**b**) EMSA analyses were used to measure interactions between proteins from K562 cells and biotin-labelled oligos of AG WT, mutated AG1 and mutated AG2 with sequences shown in **a**. Two DNA–protein complexes were detected with AG WT oligos, but only one complex was detected for AG1 mutant oligos (lower motility—interaction at the AG2 site) and AG2 mutant oligos (higher motility—interaction with the AG1 site). Arrowheads point to specific activities termed AG1 and AG2 and to free oligonucleotides. (**c**) Specificity of AG1 complex formation. Biotin-labelled double-stranded AG2-mutated oligonucleotides, which contain an intact AG1 site and a mutated AG2 site, interacted with K562 nuclear protein extracts in the presence and absence of specific competing unlabelled oligonucleotides (AG2) and nonspecific competing unlabelled oligonucleotides (AG mut, which could not participate in either AG1 or AG2 complexes). Increasing concentrations of unlabelled AG2, but not AG mut, competed for the AG1 complex. The molecular ratios of specific competitor and probe were 1:1, 1:10 and 1:100, and unspecific competitor and probe were 1:1 and 1:100. The ‘+' symbol indicates that the reagent was added to the binding reaction, whereas the ‘−' symbol indicates that the reagent was not included.

Previous studies have shown that the Rep-P replicator at the *HBB* locus (Fig. 1a) contains a 45-bp asymmetric purine:pyrimidine or an AG-rich (AG) region essential for its replicator and anti-silencer functions^35^. We reasoned that proteins capable of binding this domain would likely play key roles in replication initiation. We used an electrophoretic mobility shift assay (EMSA) to detect AG-binding activities. When oligonucleotides matching the AG domain were incubated with nuclear protein extracts from K562 cells, two shifted DNA–protein complexes were identified (Fig. 1b), indicating that at least two protein complexes (here termed AG1 and AG2, with the AG1 complex demonstrating the faster mobility) could bind to AG *in vitro*.

We performed intensive mutagenesis of the 45-bp AG wild-type (WT) oligo to identify the specific protein-binding sites (Supplementary Fig. 1a). We found that nucleotide substitutions G10T and G12T eliminated the AG1 complex, while nucleotide substitutions T28G and G30T disrupted the AG2 complex (Fig. 1b and Supplementary Fig. 1a). The substitutions that disrupted the AG1 complex replaced two guanines and also disrupted a potential G-quadruplex at the asymmetric domain, but other substitutions that eliminated G-quadruplexes (for example, GQM, GQEM variants, Supplementary Fig. 1a) did not affect AG1 binding. Specific competitors, but not mutant competitors, could eliminate the observed mobility shifts in a dosage-dependent manner, suggesting that those mobility shifts represented sequence-specific DNA–protein interactions (Fig. 1c, compare lanes 3–6 with AG1 competitor with lanes 7–9 with a nonspecific competitor). AG-binding activities could be detected in non-erythroid cell lines including human T-cell leukaemia (Jurkat) cells^45^ and human colorectal carcinoma (HCT116) cells (Supplementary Fig. 1b). These observations are in line with previous *in vivo* footprinting analyses^46^, which revealed protection at the sites corresponding to G10T and G12T (termed AG1) and T28G and G30T (termed AG2).

Since the AG sequence is essential for replication initiation at Rep-P sites^34^,^35^, we set out to find the protein or proteins that interacted with AG1 and/or AG2 as we reasoned that those proteins likely play an important role in regulating replication initiation at Rep-P. In a separate study^46^ we identified proteins that bound the AG2 site and demonstrated that their functions were related to gene expression. In the current study, therefore, we have concentrated on the AG1 site-binding proteins.

We performed a modified biotin pull-down assay to enrich for AG1-binding proteins (Supplementary Fig. 2a). Oligonucleotides with the AG1 site mutated were added to the assay as competitors to minimize nonspecific DNA-binding and AG2-binding proteins. The resulting protein samples were sequenced using mass spectrometry (Tandem MS/peptide mapping). Of the proteins identified in the initial screen, we used the CellMiner tool^47^ to select a group of 10 proteins expressed in the K562 cell line that exhibited a high level of coexpression with replication-associated genes (Supplementary Table 1) based on the expression patterns of those genes across the NCI-60 cancer cell collection (see Methods). We then used antibodies against members of the selected group in EMSA assays to screen for a protein that binds the AG oligonucleotides but not AG oligonucleotides harbouring the AG1 mutation. We have identified one such protein candidate RepID, also known as the DCAF14, member of the Ddb1- and Cul4-associated factor family, and as PHIP, a Pleckstrin Homology domain-Interacting Protein.

**Figure 2:**
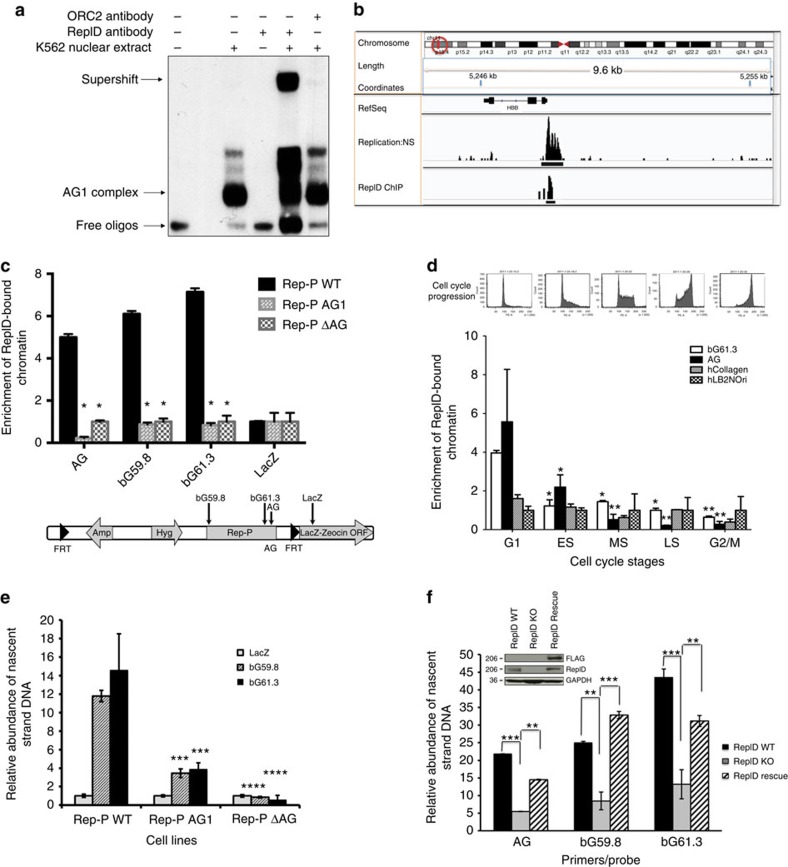
RepID interacts with replication-initiation sites. (**a**) An antibody against RepID supershifted a DNA–protein complex. EMSAs in K562 nuclear protein extracts are shown in the presence and absence of the indicated antibodies. (The additional band above the AG1 complex is not specific.) (**b**) ChIP-Seq analysis of RepID binding the *HBB* locus. Top row, a chromosome map. Below the map, First row, nascent strands reads obtained from K562 cells, second row, RepID ChIP-Seq reads obtained from K562 cells aligned to the indicated region (see ‘Methods' for details). (**c**) ChIP analysis of RepID binding in Simian CV-1 cells harbouring Rep-P variants inserted by site-specific recombination into a constant site. Rep-P WT, unmutated Rep-P; Rep-P AG1, Rep-P carrying the AG1 mutation; Rep-P ΔAG, Rep-P with the entire AG domain deleted. The LacZ primer/probe served as a negative control. All data were normalized versus amplification by the LacZ primer/probe. FRT, the fLP recombinase target (FRT) site; Amp, ampicillin; Hyg, hygromycin; LacZ-Zeocin ORF, the LacZ-Zeocin open reading frame. Statistical significance (—*P*<0.05) was calculated (*t*-test) versus Rep-P WT. (**d**) ChIP of RepID binding in K562 cells at different phases of the cell cycle. Primers and probe: bG59.8, bG61.3 and AG from the human Rep-P, hCollagen (human collagen VI), hLB2Nori (a non-initiating sequence near the LMNB2). Statistical significance (**P<0.01 or *P<0.05) was calculated versus G1. (**e**) The abundance of *HBB* sequences in nascent DNA strands from CV-1 cells harbouring Rep-P variants as described in the legend to **c**. Data were normalized to sequences amplified by the LacZ primer/probe. Statistical significance (****P<0.001 or ***P<0.005) was calculated versus Rep-P WT. (**f**) Depletion of RepID prevented replication initiation at beta-globin origin, but initiation was restored by re-introducing RepID. The abundance of sequences from the *HBB* locus was measured in nascent DNA strands isolated from U2OS cells harbouring RepID siRNA or expressing of pCMV-RepID-3 × FLAG. Statistical significance (***P<0.005 or **P<0.01) was calculated as indicated. RepID expression levels were detected using indicated antibodies as shown. Each chart in **c**–**f** shows results from a representative experiment (*n*=3).

To test the specificity of RepID antibodies, we have depleted RepID from 2,451 13T melanoma cells^48^, which are known to overexpress RepID^27^. Antibodies directed against RepID recognized a single 206-kDa protein on an immunoblot of total proteins from 2,451 13T melanoma cells, but not in cells with doxycycline-mediated induction of a short hairpin RNA (shRNA) directed against RepID (Supplementary Fig. 2b). As shown in Supplementary Fig. 2c, RepID depletion resulted in sharply decreased binding. We have further observed that antibodies directed against RepID were able to supershift the protein–DNA complex with AG-containing oligonucleotides in EMSA assays (Fig. 2a) but that the complex could not be supershifted using control antibodies, including prebleed (IgG) and antibodies directed against pre-replication complex component ORC2 (Supplementary Fig. 2d and Fig. 2a). Using purified RepID fragments expressed in *E. coli*, we identified the AG interaction domain within amino acids 923–1,126 on the RepID sequence (Supplementary Fig. 2e). These observations suggested that RepID was an AG1 site-specific binding protein and was required to form the AG1 complex *in vitro*.

To investigate whether RepID could bind the asymmetric region (corresponding to the AG oligonucleotide) *in vivo*, we used chromatin immunoprecipitation (ChIP) followed by sequencing (ChIP-Seq) to detect the binding of RepID at Rep-P (Fig. 2b in K562 and Supplementary Fig. 2f in U2OS cells; original blots are shown in Supplementary Fig. 7). In all examples of ChIP-Seq alignments (for example, Fig. 2b), the top track shows an ideogram of a portion of the relevant chromosome. The region of interest is marked with a circled box. The chromosomal coordinates of the analysed regions are shown beneath the ideogram. The RefSeq alignment of the region of interest is shown below the coordinates. The top experimental track (right below the RefSeq alignment) represents nascent-strand profiles (Replication: NS). RepID ChIP patterns (RepID ChIP) are shown below the replication profiles. As shown in Fig. 2b, replication-initiation patterns aligned with RepID binding at the *HBB* locus (human chromosome 11). ChIP assays employing real-time PCR confirmed that RepID-bound chromatin was enriched at bG61.3 (a sequence at the 3′ end of Rep-P), the Rep-P AG asymmetric region and the *HBB* locus control region (LCR; Supplementary Fig. 3a; see Supplementary Table 2 for a list of primers and Supplementary Table 3 for a list of cell lines used in the study). LCR is required for both transcription and initiation of DNA replication at the *HBB* locus^35^,^38^,^46^,^49^ and is known to interact directly with Rep-P^44^,^46^,^50^.

To evaluate the sequence specificity of RepID binding, we introduced the AG1 mutations into Rep-P by site-directed mutagenesis, and then used site-specific recombination to generate CV-1 simian cells and murine erythroleukaemia (MEL) cells that carried either Rep-P WT or Rep-P AG1 mutant transgene cassettes (designated as Rep-PWT and Rep-PAG1) at constant genomic locations^31^,^34^. This way, all mutants were analysed at identical sites to neutralize chromosomal position effects. Since integration at the MEL cells exhibited orientation-specific gene silencing^35^, we selected transgenes in which the Rep-P variants were inserted in the permissive orientation that was not prone to transcriptional inactivation. We then analysed RepID binding to these sequences at the ectopic sites. ChIP analyses with antibodies directed against RepID indicated that RepID bound to the AG region of Rep-P WT, but not to the Rep-P AG1 mutant in either simian (CV-1) cells or murine cells (MEL; Fig. 2c and Supplementary Fig. 3b, respectively).

We then tested whether Rep-P–RepID interaction occurred throughout the cell cycle; ChIP experiments were performed. Asynchronous cells were fractionated by centrifugal elutriation and the cell cycle phases (G1, Early S, Middle S, Late S and G2/M) of the fractions were determined by propidium iodide staining followed by fluorescence-activated cell sorting (FACS) analysis (top portions of Fig. 2d and Supplementary Fig. 3c). In K562 cells, which replicate the beta-globin locus in early S phase, RepID binding was restricted to the G1 and early S phases of the cell cycle (Fig. 2d). RepID binding occurred in the G1 and mid-S phases in lymphoma cells, which replicate the beta-globin locus later during the S phase (Supplementary Fig. 3c).

### Preventing RepID–origin interactions reduced initiation

We next sought to determine whether sequence mutations that affected RepID binding would also affect replication initiation. The abundance of nascent strands DNA at the Rep-P region inserted in the CV-1 system was measured by real-time PCR (Fig. 2e). High levels of nascent DNA strands located at Rep-P indicated efficient replication initiation at this site. As a negative control, we used Rep-P ΔAG, a Rep-P variant that lacked its entire AG domain and was not a functional replicator^35^. The GQEM mutants (Supplementary Fig. 1) that could not form G-quadruplexes but retained the ability to bind RepID was able to initiate replication^46^. We found that nascent DNA abundance of Rep-P WT sequences was higher than that of Rep-P AG1 mutants inserted at the same location. As expected, the control Rep-P ΔAG did not initiate replication. These data suggest that the AG1 complex is essential for efficient replication initiation within Rep-P.

We used CRISPR (clustered regularly interspaced short palindromic repeats) -Cas9 to stably deplete (‘knockout') RepID in HCT116 cells. We tested the knockout efficiency using immunoblotting against RepID antibody (Fig. 2f, inset). Nascent DNA abundance of Rep-P in RepID-depleted cells was significantly reduced. This reduction was prevented when we transiently overexpressed Flag-tagged RepID in the knockout cells (Fig. 2f). By contrast, RepID deficiency did not affect nascent DNA abundance at another origin located within the DBF4 locus (Supplementary Fig. 3d). This result supported the hypothesis that RepID plays a role in facilitating replication initiation at some but not all origins.

### Genome-wide colocalization of RepID with initiation sites

We used ChIP-Seq to assess whether a FLAG-tagged species of RepID associated with genome-wide replication-initiation events in U2OS cells. In this analysis, RepID-binding peaks called with a genomic DNA control from the same cells identified 24,222 RepID-binding sites. We found that 82.3% of RepID-binding sites localized within 2 kb of replication-initiation sites, whereas 15.4% of replication-initiation sites localized within 2 kb of a RepID-bound region. (The cutoff at 2 kb was based on the size of the isolated nascent strands, which ranged between 0.5 and 1 kb as described in the Methods section.) When this analysis was expanded to consider a 5-kb distance, 86% of RepID-bound regions (20,841 of the total 24,222 sites) colocalized with replication-initiation events and 20.7% of replication-initiation sites colocalized with RepID-bound regions. With the same analysis window, 34.4% of RepID-bound regions colocalized with transcription start sites, a distribution similar to the reported distribution of replication-initiation sites^4^. ChIP-Seq experiment using an endogenous antibody against RepID in K562 cells (Supplementary Fig. 4a) also suggested that replication-initiation events were highly enriched in RepID-binding sites.

The replication-initiation ratio, reflecting the enrichment in replication-initiation events, was the highest observed, thus far, for any DNA-binding protein including transcription factors that were previously shown to associate with the initiation of DNA replication. As shown in the detailed analyses in Supplementary Fig. 4b,c, genome-wide enrichment for replication-initiation events in K562 cells was highest at the locations of RepID binding and diminished with distance from RepID-binding sites. Replication-initiation events also colocalized to a lower extent with c-Jun-binding sites, as reported previously^4^,^51^, but did not colocalize with other transcriptional regulators (for example, SIRT6, which exhibited similar colocalization to replication-initiation peaks and simulated randomized peaks).

**Figure 3:**
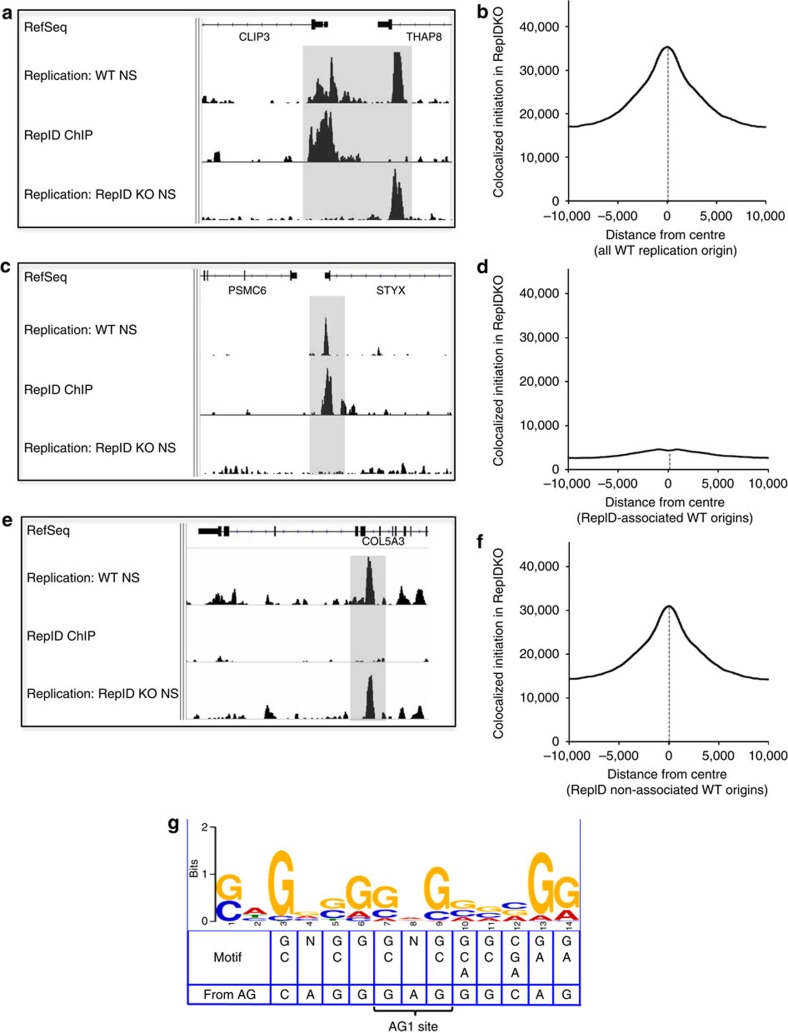
Genome-wide colocalization of RepID with replication-initiation sites. (**a**) A screenshot of a sample genomic region showing replication-initiation profiles (NS-Seq) and protein-binding (ChIP-Seq) data. Top track, below the RepSeq genes: nascent-strand patterns from cells with WT RepID (Replication: WT NS). Middle track: ChIP-Seq patterns in WT cells (RepID ChIP). Lowest track: nascent-strand patterns from cells depleted of RepID (Replication: RepID KO NS). The shaded region delineates a RepID-binding origin adjacent to an origin that does not bind RepID. An expanded screenshot of the same region is shown in Supplementary Fig. 4d. (**b**) The distribution of replication-initiation events in RepID KO cells (overall, 58,656 NS-Seq peaks) that colocalized with initiation events in WT cells, plotted as a function of the distance from the centre of WT origins (overall, 78,859 NS-Seq peaks). (**c**) A screenshot of a sample genomic region showing replication-initiation profiles as in **a**. The shaded region delineates a replication origin that binds RepID in WT cells and does not initiate replication in KO cells. An expanded screenshot of the same region is shown in Supplementary Fig. 4e. (**d**) The distribution of replication-initiation events in KO cells plotted as a function of the distance from the centre of origins that were bound by RepID in WT cells (14,716 NS-Seq peaks). (**e**) A screenshot of a sample genomic region showing replication-initiation profiles as in **a**. The shaded region delineates a replication origin that does not bind RepID in WT cells and initiates replication in both WT and KO cells. An expanded screenshot of the same region is shown in Supplementary Fig. 4f. (**f**) The distribution of replication-initiation events in KO cells along genomic regions flanking replication origins that initiated replication but did not bind RepID in WT cells (64,143 peaks). (**g**) Consensus sequence for RepID binding identified using a subset of RepID-bound regions. Consensus is aligned with a 12-bp motif, which matches the AG1 site. Data showing the abundance of the motif in RepID-bound regions or randomized files are presented in Supplementary Table 4.

We next asked whether RepID binding affected initiation activity. To that end, we measured the frequency of replication initiation along with RepID ChIP-Seq in cells with unaltered RepID and in cells that were subjected to CRISPR-mediated RepID depletion. Examples of screenshots aligning nascent-strand profiles and ChIP-Seq analyses are shown in Fig. 3a,c,e and in Supplementary Fig. 4d–h. In all screenshots, the top, middle and bottom Integrated Genome Viewer (IGV) tracks (right below the RefSeq alignment) represent replication-initiation profiles in RepID-proficient cells (Replication: WT NS), RepID ChIP-Seq patterns (RepID ChIP) and replication-initiation profiles in RepID-depleted cells (Replication: RepID KO NS), respectively. As shown in Fig. 3a (also Supplementary Fig. 4d,g), RepID bound replication origins and replication origins that were not bound by RepID could be found throughout the genome, and could sometimes be located at adjacent regions. To determine the effect of RepID depletion on replication initiation, we next plotted the genome-wide frequency of initiation in RepID KO cells for all 20-kb genomic regions flanking replication origins in RepID WT cells (including those that both were and were not bound by RepID). We observed replication-initiation events in both WT and KO cells (Fig. 3b). We then plotted the genome-wide initiation frequency solely in RepID-bound origins (identified by RepID binding in WT cells—for examples, see Fig. 3c and Supplementary Fig. 4e,h). In this subgroup of origins, the frequency of initiation in KO cells was very low, with no notable colocalization (Fig. 3d). Conversely, replication origins that were not associated with RepID in WT cells (for example, see Fig. 3e and Supplementary Fig. 4f,g) were able to initiate replication in both RepID WT and KO cells (Fig. 3f). These observations suggested that RepID was present at a subset of replication origins and was essential for initiation, specifically at those origins.

We next asked whether RepID-binding sites shared common sequence motifs. First, we identified a subset of RepID-bound regions for further analysis. This subset of RepID-bound regions was required to span a region shorter than 400 bp, to be located more than 1 kb away from a neighbouring RepID-bound region and to have a peak score greater than or equal to 150. We have identified 268 RepID-bound regions that fit these criteria. A MEME-ChIP analysis for *de novo* motif (http://meme-suite.org/tools/meme-chip) identified several motifs. The top motif that was output by MEME-ChIP was truncated to a 12-bp sequence that matched a segment of the AG region from the Rep-P replicator, which contains the AG1 site (Fig. 3g).

To ask whether this motif was enriched within the RepID-binding sites in the context of the entire genome, we compared the subset of 268 RepID-bound regions to three randomized files, each containing 268 sequences from random genomic loci that were of the same length and GC content as the sequences in the original file. As seen in Supplementary Table 4, 71.64% of the RepID-bound regions contained this motif, while 16–22% of the randomized sequences contained this motif. As expected, 64.18% of these RepID-bound regions were within 2 kb of a replication origin, whereas only 20–25% of the randomized sequences were within 2 kb of a replication origin. Consistent with a significant but partial overlap of the motif with replication origins, this sequence was found at the *HBB, CTCF, JunB* origins, but not at the *DHFR o*rigin (Fig. 2b and Supplementary Fig. 4g–i). Of those sequences in each file that were nearby a replication origin, 70.35% of the RepID-bound regions and 30–43% of the regions in the randomized files contained the motif. The high association in the randomized files most likely reflected the fact that the randomized origin regions contained a high frequency of GC-rich sequences. RepID regions that were not associated with replication origins exhibited 71.88% association with the motif, and randomized non-origin sequences exhibited between 12 and 16% association. These observations are consistent with a role for the motif in facilitating RepID binding but not in the decision to initiate DNA replication.

### Abnormal DNA replication in *RepID*-deficient MEFs

**Figure 4:**
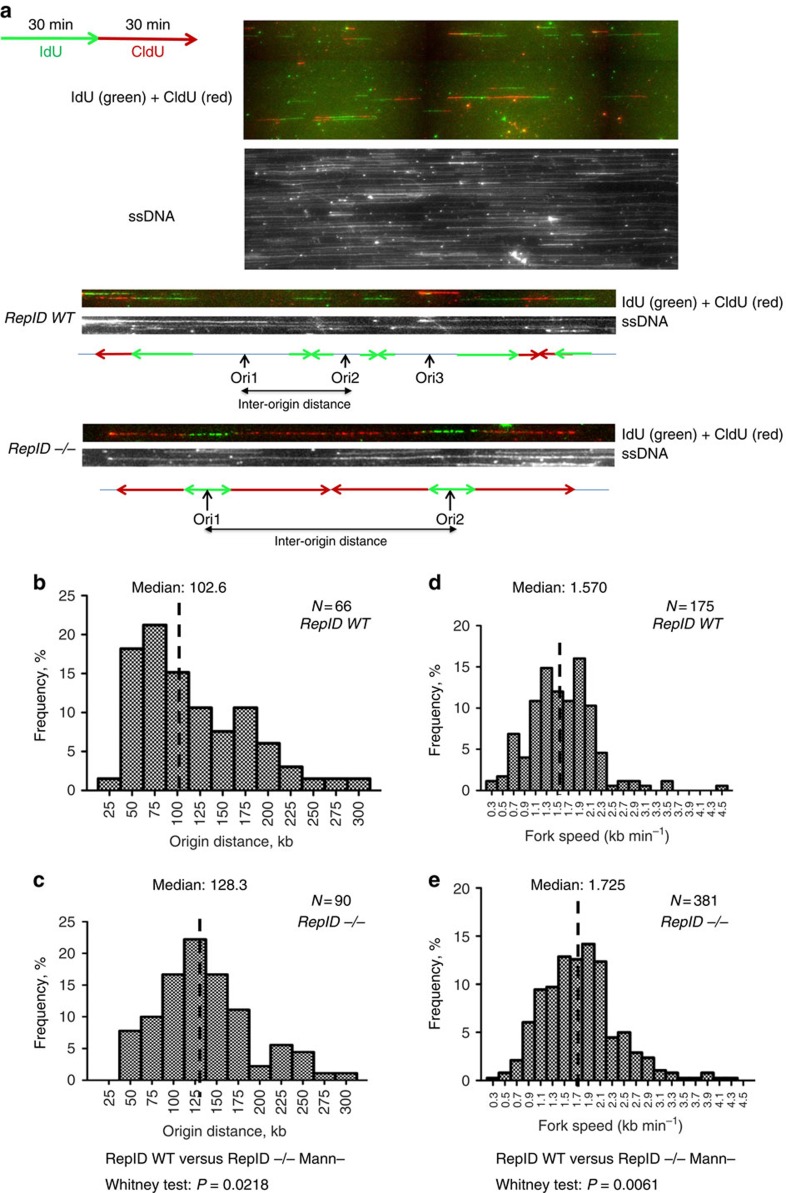
Depletion of RepID decreases the frequency of replication-initiation events. (**a**) Cells were sequentially labelled with IdU followed by CldU. Top panel, a typical field with replication signals (IdU detected in green and CldU detected in red). Second panel, the same field with all fibres labelled with an antibody detecting single-strand DNA (ssDNA; grey). Third and fourth panels, an example of CldU-IdU (third) ssDNA (fourth) fibre tracks from *RepID WT* MEFs. Fifth and sixth panels, an example of CldU-IdU (fifth) ssDNA (sixth) fibre tracks from *RepID −/−* MEFs. Illustrations of replication fork patterns are shown below the ssDNA track. The lengths of fibres label associated with ldU and CIdU incorporation and inter-origin distances were measured (see Methods), and rates of replication fork progression were calculated based on these values. Ori, origin; ssDNA, DNA detected by anti-single strand antibody. (**b**,**c**) Measurements of the distribution of distances between replication origins in DNA fibres from *WT* MEFs and *RepID −/−* MEFs. (**d**,**e**) Measurements of the distribution of replication fork progression rates for *WT* and *RepID −/−*MEFs. The differences between measurements from fibres obtained from wild-type and *RepID*-deficient MEFs were significant at *P*<0.05 (*P*=0.0218 for inter-origin distance and *P*=0.0061 for replication fork speed as calculated using the Mann–Whitney test). Normality test by Kolmogorov–Smirnov test showed that the distributions of data for **b**–**e** are not normal (*P*<0.01).

We used single fibre analyses (DNA combing) to determine the genome-wide consequences of RepID depletion. When replication patterns in *RepID WT* and *RepID −/−* murine embryonic fibroblasts (MEFs)^52^ were compared, we observed differences in replication fork speed and distance between replication origins (Fig. 4). In *RepID WT* cells, the median distance between origins was 102.6 kb, and the median fork speed was 1.570 kb min^−1^ (Fig. 4a,b,d). In *RepID −/−* cells, the median distance between replication origins was 128.3 kb, and the median fork speed was 1.725 kb min^−1^ (Fig. 4a,c,e). In *RepID*-deficient cells, therefore, there were significantly fewer replication-initiation events (compared with *WT* cells). This reduction in initiation events was associated with a compensatory increase in replication fork speed. Although replication origin distances were longer in *RepID*-deficient MEFs, *RepID*-deficient MEFs continued to initiate replication, consistent with the fact that those cells progressed through the S phase and that *RepID*-deficient mice were viable. We hypothesize that the small differences in inter-origin distance we observed reflected the fact that other origins would compensate for initiation deficiency, as previously reported^53^. Despite the small differences, notably the differences between replication profiles in *RepID WT* and *RepID −/−* MEF cells were statistically significant (*P*-values are 0.0218 and 0.0061 for inter-origin distance and fork speed, respectively).

**Figure 5:**
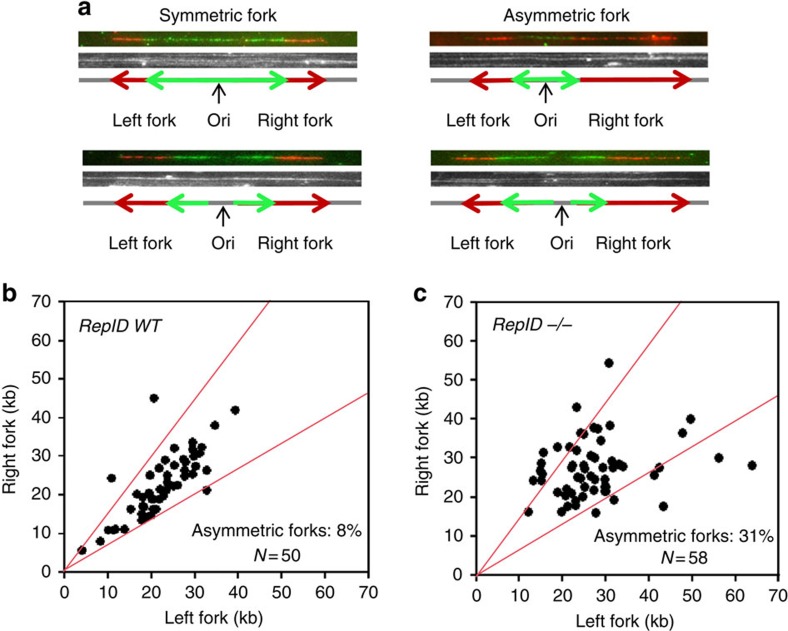
*RepID*-deficient MEFs exhibit replication fork asymmetry. (**a**) Examples of DNA fibres derived from wild-type and *RepID −/−* MEFs that contain symmetric and asymmetric replication forks. (**b**,**c**) Scatter plots of left and right fork lengths in RepID *WT* (**b**) and *RepID −/−* (**c**) cells. The percentages of the asymmetric forks (outside the red lines) and the number of replication forks measured in both cells are presented on the plots, demonstrating that 31% of forks were asymmetric in *RepID−/−* MEFs, whereas 8% of forks were asymmetric in *RepID WT* MEFs.

We also assessed the extent of stalled replication, measured as the frequency of asymmetric replication forks, in *RepID WT and RepID−/−* cells (Fig. 5). We defined a replication fork as asymmetric if one side of the fork was 33% longer than the other side (for example, see Fig. 5a). We frequently observed asymmetric replication forks in *RepID−/−* cells (31% compared with 8% in *RepID WT* cells), suggesting that these cells experienced frequent replication fork stalling (Fig. 5b,c). The percentage of DNA fibres exhibiting a replication signal provides another indication of replication activity. In *RepID−/−* cells, a significantly lower fraction of the DNA fibres (7.86%) exhibited a replication signal than fibres from *WT* cells (15.6%, Supplementary Fig. 5a), consistent with a decreased proliferation rate previously reported in *RepID*-deficient cells^26^. FACS analyses indicated that *RepID−/−* cell cultures had fewer cells in the S phase (and G2/M phase) than *WT* cells (Supplementary Fig. 5b,c). These observations suggested that, in addition to the observed low initiation rate during the S phase, RepID deficiency might also result in a lower frequency of cells entering the S phase. Taken together, our data demonstrated that RepID was required for proper initiation of DNA replication and proper elongation of replication forks. In the absence of RepID, DNA replication initiation and cell cycle progression were both partially impaired.

### RepID participates in a distal Rep-P interaction with LCR

**Figure 6:**
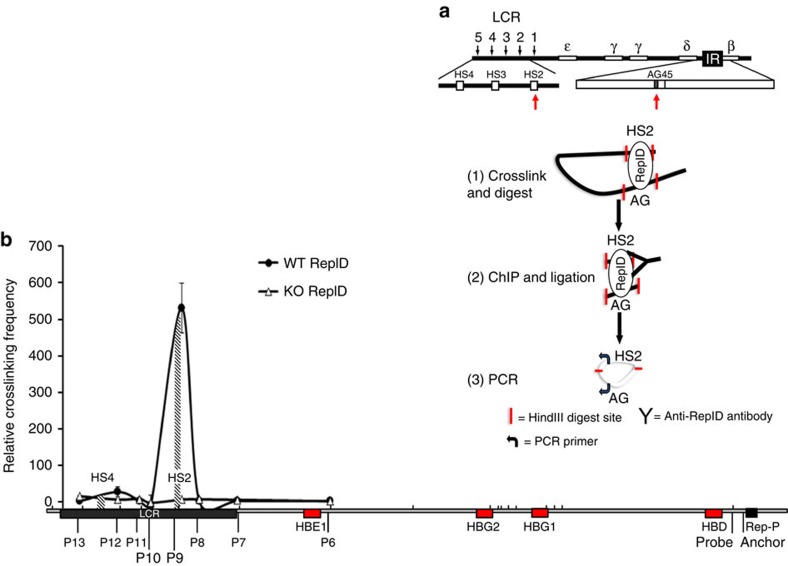
RepID is present in a complex between LCR and Rep-P in early replicating *HBB* loci. (**a**) Schematic illustration of the beta-globin locus and the outline of ChIP-3C procedure. Cells were lysed and digested with HindIII. Crosslinked chromatin fragments were immunoprecipitated with anti-RepID antibody and ligated. Crosslinks were reversed and DNA was isolated and amplified by PCR. The primers and TaqMan probe used for the ChIP-3C analysis are listed in the ‘Methods' section. The red arrows indicate the location of AG and HS2 on the beta-globin locus, which are more than 50 kb away from each other. (**b**) ChIP-3C analysis of long-distance RepID-associated chromatin interactions at the *HBB* locus in U2OS WT and RepID knockout cells. The 3C primers correspond to sequences near the downstream sticky ends of the 3C fragments. Primer/probe combinations were designated p6 to p13, and their locations are indicated as half arrows. The *x* axis shows the positions of the restriction fragments on the genomic scale (vertical bars). The diagram represents the extent of PCR amplification of each primer/probe pair. Values represent the average of triplicate samples (bars represent s.e.'s). Data were obtained after subtraction of no ligation controls and were normalized to the AG site loading control. Primer efficiencies were normalized using a single bacterial artificial chromosome (BAC) clone covering the genome segment under study.

We used ChIP-chromosome conformation capture (ChIP-3C) to investigate whether RepID associated with LCR sequences that directly interacted with Rep-P at the *HBB* locus. In this procedure, crosslinked RepID-bound chromatin was isolated, digested with a restriction enzyme (HindIII) and re-ligated with T4 ligase. With this procedure, if two distant *cis*-elements are interacting with each other, the ligation will link the two sequences together (Fig. 6a). As shown in Fig. 6b, we were able to amplify a DNA fragment when we amplified crosslinked RepID-bound chromatin with a primer from the HS2 site of the *HBB* locus control region and another primer from Rep-P. Amplification using the Rep-P anchor with other primer pairs spanning the locus exhibited significantly lower amplification values, suggesting that the procedure identified an interaction between HS2 and Rep-P in RepID-bound chromatin (Fig. 6a). As expected, this interaction was not observed when we used chromatin from RepID-depleted cells (RepID KO). ChIP-3C of RepID-bound chromatin from K562 cells yielded a 139-bp PCR product amplified with Rep-P and HS2 primers (Supplementary Fig. 6a). To test whether the amplified fragment indicated an interaction between Rep-P and HS2, we cloned and sequenced the amplification product. As expected, the amplified fragment contained both HS2 and Rep-P sequences linked at a HindIII site (Supplementary Fig. 6a). Chromatin loops were detected at both HS2 and HS4 within the *HBB* locus in K562 cells (Supplementary Fig. 6b). Interactions between HS4 and Rep-P were not observed in RepID-associated chromatin (compare Fig. 6b with Supplementary Fig. 6b)^54^. Since immunoprecipitation with an antibody directed against RepID detected the HS2–Rep-P interaction in both K562 and U2OS cells, our results suggest that the *HBB* locus in both erythroid and non-erythroid cells exhibits a RepID-associated interaction between LCR and Rep-P at HS2.

## Discussion

In this study, we report that the RepID protein binds distinct mammalian replication origins and is required for sequence-specific initiation of DNA replication at these origins. RepID was first identified by its interaction with a single replicator sequence, which is essential for replication initiation at the *HBB* locus. RepID exhibits genome-wide enrichment at replication-initiation sites. Cells depleted of RepID exhibited diminished initiation frequency, slower elongation of replication forks and frequent replication fork-stalling events. Together, these observations support the notion that distinct DNA–protein interactions at specific groups of replicators dictate replication initiation, and that RepID is a mediator of such interactions.

Although mammalian replicators can initiate DNA replication at ectopic sites, there is an ongoing debate whether replication-initiation sites are determined by specific DNA sequences or solely by chromatin structures^1^,^3^,^10^. Chromatin modifications, for example, dimethylation of histone H3 lysine K79 (H3K79Me2), can associate with a distinct fraction of replication-initiation sites in the human genome, and can mark replicated chromatin during the S phase to prevent re-replication and preserve genomic stability^55^. The lack of binding specificity by components of pre-replication complexes suggests that, in addition to the requirement for those complexes to initiate DNA replication, distinct replicator-interacting proteins might be required to initiate DNA replication in a sequence-specific manner at particular loci^3^,^56^. Our results suggest that RepID is one such protein that interacts with a subgroup of origin sequences.

**Figure 7:**
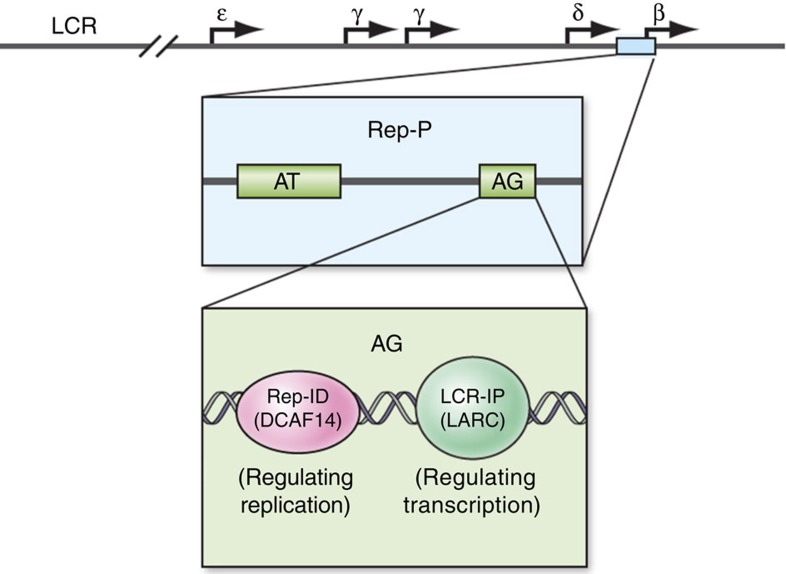
Spatial organization of DNA–protein interactions within Rep-P. The asymmetric region within the Rep-P replication of the *HBB* locus is involved in two distinct DNA–protein interactions. An interaction with the LARC complex facilitates transcription and maintains an open chromatin conformation in erythroid cells^46^, whereas the interaction of adjacent sequences with RepID facilitates the initiation of DNA replication from Rep-P. Top diagram, a schematic of the *HBB* locus, illustrating the location of the Rep-P replicator; middle, a schematic of the Rep-P replicator, illustrating the location of the AG sequence (sequence shown in Fig. 1a); lower diagram, two adjacent protein–DNA interactions within AG.

The locations and timing of replication-initiation events are often affected by interactions with *cis*-acting distal genomic elements^1^,^3^,^19^,^54^,^57^ including promoters, enhancers and insulators. These interactions can form chromatin loops to determine where and when replication initiates and likely coordinate replication with transcription. At the human *HBB* locus, interaction of Rep-P with the LCR is essential for initiation of DNA replication^49^. As summarized in Fig. 7, the RepID-binding site at the *HBB* locus is adjacent to the binding site of the LCR-associated remodelling complex (LARC), which regulates transcription^46^. Despite the close proximity of transcription and replication complexes at the AG element, RepID is a sequence-specific replicator-interacting protein that does not act as a transcription factor at the *HBB* locus, as the antisilencing activity of the AG element is not affected by mutations that prevent RepID binding^46^. Consistent with a separation of function between the two AG-binding proteins, AG2 mutations, which prevent LARC binding^46^, do not prevent initiation of DNA replication, and prevention of RepID binding does not affect transcriptional activity. These observations rule out a possible causal relationship between RepID and LARC binding. The involvement of RepID in the interaction between Rep-P and LCR suggests a possible mechanism for dictating replication-initiation events. RepID may thus play a role in coordinating transcription and replication at the *beta-globin* locus and similar RepID-binding replicators through its tandem bromodomains.

We have identified a common GC-rich motif for RepID-binding, which is evident in the AG region of the Rep-P replicator and resembles motifs previously identified for replication-initiation sites in mice and *Drosophila*^7^. Although this motif is GC-rich, the ability to bind RepID is distinct from the ability to form G-quadruplex structures^18^ or other motifs that were associated with many replication origins^7^,^51^. Indeed, RepID-bound regions contained this motif whether they contributed to replication initiation or not. These studies support the notion that RepID is a sequence-specific DNA-binding protein that contributes to replication initiation at a subset of replication origins; however, other factors such as histone modifications and differentiation state might also affect the frequency of initiation.

RepID is a member of the DCAF family of proteins that interact with Cullin-RING-based E3 ubiquitin ligases^28^. WD40 repeat-containing proteins, including RepID (DCAF14) BRWD3 and BRWD1 (DCAF19), have diverse functions in eukaryotes that are often associated with cell cycle progression. Examples include LRWD1/ORCA facilitating ORC-binding to chromatin^22^, RFWD3 associating with replication protein A following damage repair^58^, DCAF2 (CDT2), mediating DNA-damage-induced p97-mediated Cdt1 proteolysis^29^ and DCAF1 (VprBP) facilitating cellular proliferation^59^. Similar to RepID, WD40-repeat-containing DCAF proteins may, therefore, act as adaptors for specific protein–chromatin interactions.

The replication deficiencies observed in the absence of RepID are consistent with the requirement of RepID for pancreatic β-cell proliferation^25^ and strongly suggest a role for RepID in the regulation of DNA replication. We observed that both the initiation and elongation steps of DNA synthesis seem affected in RepID-deficient MEFs, which initiate DNA replication at a low frequency and exhibit frequent replication fork stalling. These data are consistent with previous observations, suggesting that replication-initiation frequencies and elongation rates are interconnected. Recent studies demonstrate that a low frequency of active replication origins (genome-wide and in fragile sites) might trigger genomic instability, and, conversely, that the pace of replication could dictate the frequency of initiation events^53^,^60^,^61^,^62^. Although it is formally possible that RepID exerts unrelated effects on initiation and elongation, the colocalization we observed between RepID and replication origins and the requirement for RepID for initiation at a group of replication origins lend support to the hypothesis that RepID deficiency reduces the frequency of initiation events and this low frequency, in turn, affects genomic stability.

Although RepID deficiency affected replication-initiation rates, notably not all replication-initiation events were disabled, suggesting that many replication-initiation events did not require RepID. Although our observations could not formally rule out an indirect role of RepID in replication (for example, by affecting the activity or facilitating transcription of replication factors that regulate replication in a subgroup of RepID-binding sites), the data are consistent with the hypothesis that RepID facilitates initiation at RepID-binding origins. It is likely, therefore, that metazoan replication origins can be divided into different categories, each associated with a specific modifier protein that determines origin usage according to cell type and developmental stage. RepID may thus be the first member of a series of proteins, which we propose to name RepIDs that interact with particular subsets of replication origins to determine replicator activity. Such proteins might facilitate interactions between the cell cycle-regulatory network and chromatin to determine where and when DNA replication starts and how replication coordinates with transcription and other chromatin transactions.

## Methods

### Cell lines and culture conditions

We grew all cells in DMEM (Invitrogen, Cat. no. 10564-011) supplemented with 10% heat-inactivated fetal calf serum in a 37 °C/5% CO_2_ incubator. We added 1% penicillin–streptomycin (Invitrogen, Cat. no. 15140-163) and 1% Fungizone (Invitrogen, Cat. no. 15290-018) to the culture media as needed. All cells tested negative for mycoplasma. The selection drug, Zeocin (Invitrogen, Cat. No. R250-01), was added to CV-1 cells to a final concentration of 100 μg ml^−1^ before plasmid transfection. For RepID knockout stable clone selection, we added puromycin (Invitrogen, Cat. No. A11138-03) at a final concentration of 0.3125 μg ml^−1^ to HCT116 cells and 1 μg ml^−1^ to U2OS cells after plasmid transfection. Mouse embryonic fibroblasts were isolated from RepID-proficient and null mice^26^. CV-1 and RL4 cells harbouring Rep-P WT or mutant Rep-P were originally obtained from American Type Culture Collection (ATCC) and modified to facilitate site-specific insertions of defined sequences by FLP-mediated recombination^31^. Melanoma cell line 2451 13T (ref. 48) was a gift from Dr Yardena Samuels (NHGRI). All other cancer cell lines were obtained from ATCC (www.atcc.org).

Cell cycle fractionation was performed by centrifugal elutriation of asynchronously growing K562 cells. The purity of the fractions was measured using flow cytometry.

### EMSA

The oligonucleotide substrates used in the EMSA assays included the 45-bp asymmetric region (AG WT), the AG1 mutant oligo and the AG2 mutant oligo (Fig. 1). For EMSA analysis, biotin-labelled forward and reverse oligonucleotides were mixed at a final concentration of 100 pM, and then boiled at 100 °C for 1 min. After annealing, the oligonucleotides were incubated with 20 μg nuclear extract in 1 × binding buffer (10 mM Tris-HCl, 2.5% glycerol, 0.05% NP-40, 25 mM KCl, 1 μg poly (dI.dC) and 1 mM dithiothreitol (DTT)) for 30 min at room temperature. Reactions were subsequently subjected to electrophoresis using a 6% DNA retardation gel (Invitrogen Cat. no. EC6365BOX) on ice for 1.5 h and then transferred to a positively charged nylon membrane and ultraviolet-crosslinked. We used the LightShift Chemiluminescent EMSA kit (Pierce, Cat. no. 20148) for biotin-labelled DNA detection. For competition assays, biotin-labelled double-strand AG2 mutant oligonucleotides were mixed at a final concentration of 100 pM with unlabelled double-strand mutant oligonucleotides at the final concentrations of 100 pM, 1 nM and 10 nM. For supershift assays, nuclear extracts were incubated with antibodies before the labelled annealing oligonucleotides were added. Antibodies included RepID (A302-055A, Bethyl Laboratories Inc.) and ORC2 (559266, BD Biosciences). Antibodies were validated by the use of specific shRNA to deplete RepID in 2451 13T melanoma cells.

### shRNA knockdown

Specific silencing of endogenous RepID was achieved using an inducible shRNA-expressing vector, pSingle-tTS-shRNA (Clontech). shRNAs were inserted into the plasmid using the XhoI and HindIII cloning sites and were delivered into 2451 13T melanoma cells^48^. Stable clones were selected, and cells conditionally expressing shRNA directed against RepID were induced (or not) with doxycycline for 16 days.

### RepID knockout stable cells by the CRISPR-CAS9 system

A 20-bp guide sequence (5′- GTGATAAAATGATCCGAGTC -3′) targeting DNA within the fourth exon of RepID was selected from a published database of predicted high-specificity protospacer-PAM target sites in the human exome. Two complementary oligos (5′- CACCGTGATAAAATGATCCGAGTC -3′ and 5′- AAACGACTCGGATCATTTTATCAC -3′) containing the RepID guide sequence and BbsI ligation adapters were synthesized by Eurofins MWG Operon (Alabama, USA). Oligo (100 mM, in a total volume of 10 ml) was annealed and ligated into the BbsI-digested pX330 vector. The sequence of the construct was verified by sequencing. For stable selection, HCT116 or U2OS cells were cultured in six-well dishes to 70–80% confluence. Cells were co-transfected with 2 mg of RepID single guide RNA (sgRNA) plasmid plus 2 mg of linearized pCR2.1 vector harbouring a puromycin-resistance gene and 10 ml of Lipofectamine 2000 (Life Technologies) per well. Twenty-four hours post transfection, 10% of transfected cells was seeding to 10-cm dishes in a serial dilution. In the next day, cells were cultured in the medium with appropriate concentration of puromycin for selection.

### Nascent-strand DNA analysis

Nascent-strand DNA^31^,^34^ was extracted from asynchronous cells. This DNA was fractionated on a neutral sucrose gradient. DNA fractions (0.5–1 kb) were collected and treated with λ exonuclease to remove non-RNA-primed genomic DNA fragments. Nascent-strand DNA was quantified with real-time PCR using an ABI 7900 thermocycler (primers and probes used for real-time PCR are listed in Supplementary Table 2). AG1 and AG2 mutations were introduced by site-directed mutagenesis^46^.

### ChIP analysis

ChIP analyses were performed with 1% formaldehyde-fixed K562, U2OS RepID 3XFlag, CV-1 and RL4 cells using the Millipore ChIP assay kit (Cat. no. 17–295). Antibodies included normal rabbit IgG (sc-2027), anti-Flag (F3165; Sigma-Aldrich) and anti-PHIP (sc-68354; Santa Cruz Biotechnologies). ChIP samples were analysed with real-time PCR using an ABI 7900 thermocycler, with primers/probes listed in Supplementary Table 2. ChIP experiments were performed on at least two biological replicates for each cell line, and PCR amplifications were performed in triplicates. All ChIP data were expressed as nanogram-amplified DNA (calculated based on standardized curves of genomic DNA) divided by the number of molecules amplified from the same preparation of ‘Input' and ‘mock' samples.

### Peak-calling of ChIP-seq and NS-seq

For nascent-strand and ChIP-Seq experiments, regions that were significantly enriched were identified using two peak-calling programmes. For RepID ChIP, the MACS peak-calling programme http://liulab.dfci.harvard.edu/MACS/ was used, comparing reads from the ChIP-seq experiment to genomic input reads from the same cell line, with default parameters and a *P*-value=1e−9. Overall, 24,222 regions enriched for RepID were identified. For U2OS nascent-strand peak-calling, the SICER programme (http://home.gwu.edu/~wpeng/Software.htm) was used. This programme was chosen because of the presence of wide initiation zones. Nascent-strand reads were called by comparison with U2OS genomic reads using a window size of 200 bp, gap size of 600 bp and false discovery rate (FDR) of 0.01. Overall, 92,814 regions enriched for replication initiation were identified. Screenshots of example genomic loci were captured using the IGV genome browser (https://www.broadinstitute.org/igv/). Intersections between two files of enriched regions were identified using a custom script (available on request). The genome-wide colocalization analyses comparing bed files of RepID-bound regions and replication-initiation sites were performed using GenomeInspector with a 20-kb window size.

### Identification of a consensus sequence

A subset of RepID-enriched regions from FLAG ChIP-Seq experiments in U2OS RepID-3 × FLAG stable cells was identified with the following criteria: enriched regions were shorter than 400 bp, located at least 1 kb away from the nearest RepID peaks, and have peak scores greater than 150. These criteria identified 268 RepID regions that were submitted to MEME-ChIP, with parameters asking for a sequence between 6 and 22 bp. The first motif was a 21-bp motif, which was truncated to a 12-bp motif that could be found in the AG sequence of the Rep-P replicator containing the AG1 site. A custom string search script was used to identify the percentage of sequences containing the motif. A custom script was used to create random files that had the same number of sequences of the same length, but from random genomic loci. (Both scripts are available on request.)

### DNA fibre analyses

DNA combing analysis of replicating DNA was performed as follows using previously published methods^63^. *RepID* WT and *RepID−/−* cells were pulse-labelled with 20 μM IdU (Sigma, Cat. no. I-7125) for 30 min, and then with 50 μM CldU (MP biomedical, Cat. no. 105478) for 30 min. Following the CldU pulse, the cells were embedded in low-melting agarose plugs, and were lysed in the plug with lysis buffer (1 mg ml^−1^ proteinase K, 50 mM EDTA, 1% N-lauroyl-sarcosine, 10 mM Tris-Cl, pH 8.0) at 50 °C overnight. After digesting the plug with β-agarase (New England Biolabs, Beverly, MA), DNA was combed to silanized surfaces (Microsurfaces Inc.). Replicating DNA was detected with anti-IdU (BD, Cat. no. 347580), anti-CldU (Accuratechmecal, Cat. no. OBT0030) and anti-single stranded DNA (Chemicon, Cat. no. MAB3034) antibodies. Images were captured using the Attovision software and the epifluorescence microscope Pathway (Becton Dickinson). For replication fork rate measurements, only internal replication signals on single-strand DNA (not colocalizing with DNA fibre bundles, not located at the end of a strand) were selected for analyses. Samples have included a BAC as a molecular marker, allowing us to assess the uniformity of fibre-stretching and convert labelled signal length (in microns) to fibre length (in kb). Signals were marked for evaluation by ‘blind' measurers (not knowing which samples they were measuring); signal length was measured using the Image J software (open source from National Cancer Institute, NIH) followed by automatic compilation of signal lengths into an Excel worksheet. Replication fork velocities were calculated using elongating fork signals only (initiating forks were eliminated). Fork velocities and origin distances were calculated using a constant of 2 kb μm^−1^. Experiments were performed at least in duplicate using independent biological isolations of DNA fibres for each experimental condition. Statistical analyses were preformed with the Prism 5.0 software (GraphPad Software Inc.) using the non-parametric Mann–Whitney rank-sum test.

### Cell cycle analysis

Cells were pulse-labelled with 20 μM 5-ethynyl uridine (EdU) for 45 min before cell harvest. EdU staining was performed using the Click-iT EdU kit (Invitrogen, C10424) according to the manufacturer's protocol. 4,6-Diamidino-2-phenylindole or propidium iodide (PI) were used for DNA staining. BD LSRFortessa cell analyser with the FACSDiva software was used for cell cycle analysis.

### Nuclear protein extract preparation

Cells were harvested and incubated in sucrose buffer containing NP-40 (320 mM sucrose, 10 mM Tris-HCl pH 8.0, 3 mM CaCl_2_, 2 mM MgOAc, 0.1 mM EDTA, 0.5% NP-40, 1 mM DTT, 0.5 mM phenylmethylsulphonyl fluoride (PMSF), protease inhibitor cocktail (Sigma, Cat. P8340) and phosphatase inhibitor cocktail (Roche, Cat. 04906845001)). Nuclei were harvested by centrifugation, washed and resuspended in low-salt buffer (20 mM HEPES pH 7.9, 20 mM KCl, 0.2 mM EDTA, 25% glycerol (v/v), 0.5 mM DTT, 0.5 mM PMSF, protease inhibitor cocktail and phosphatase inhibitor cocktail). And then an equal volume of high-salt buffer (20 mM HEPES pH 7.9, 800 mM KCl, 0.2 mM EDTA, 25% glycerol (v/v), 1% NP-40, 0.5 mM DTT, 0.5 mM PMSF, protease inhibitor cocktail and phosphatase inhibitor cocktail) was added. The suspension was incubated at 4 °C for 45 min with rotation, and then were centrifuged it at 14,000 *g* for 15 min at 4 °C. The supernatant was the nuclear soluble fraction, and the pellet was the insoluble chromatin-bound fraction. Before use, protein concentrations were determined with a Bio-Rad DC protein assay kit (Bio-Rad, Cat. no. 500-0112).

### Biotin pull-down assay and mass spectrometry

The forward and reverse strands of biotin-labelled AG WT oligos (2.5 μg each) were mixed and incubated for 10 min at room temperature, and then 15 ml 1 × binding buffer (10 mM Tris-HCl, 2.5% glycerol, 0.05% NP-40, 25 mM KCl, 1 μg poly (dI.dC), 1 mM DTT and proteinase inhibitor cocktail) was added. And then 0.5 ml of the Dynabeads M-280 Streptavidin (Invitrogen, Cat. no. 112.05D), 250 μg of the unlabelled competitor AG1 oligo mixture and 500 μg of K562 nuclear protein extract were added. The Dynabeads were collected by using a magnet after 2 h of incubation at room temperature. After washing the beads with 1 × binding buffer for three times, the proteins were eluted by adding 40 μl of 2 × SDS sample buffer and ran on a 4–12% Tris-Glycine SDS–PAGE gel with coomassie-blue. Specific bands were sent for mass spectrometry analysis at the Advanced Technology Program of the National Cancer Institute at Frederick. Of the 117 proteins identified in the initial screen, proteins identified based on a single peptide, low Mascot scores and known contaminants such as keratin were eliminated, and a top group of 10 proteins was selected for further analysis using the CellMiner tool (http://discover.nci.nih.gov; see below for gene expression analyses).

### Gene expression pattern analysis

Gene expression data used in the current study can be accessed at CellMiner, at http://discover.nci.nih.gov^47^ or from the GEO data repository (accession numbers GSE22821, GSE5846, GSE5949, GSE5720 and GSE32474). To obtain the transcription profile, the NCI-60 cancer cell lines were obtained from the Developmental Therapeutics Program, Division of Cancer Treatment and Diagnosis^64^. Expression levels for transcripts were determined with data for probes from five platforms and normalization (http://discover.nci.nih.gov/cellminer/). Affymetrix (Affymetrix Inc., Sunnyvale, CA) Human Genome U95 Set (HG-U95); the Human Genome U133 (HG-U133); the Human Genome U133 Plus 2.0 Arrays (HG-U133 Plus 2.0); and the GeneChip Human Exon 1.0 ST array (GH Exon 1.0 ST) were included. Agilent (Agilent Technologies Inc., Santa Clara, CA) Whole Human Genome Oligo Microarray was also included. Composite probe set intensity values were transformed to *z*-scores through subtraction of their 60 cell line means and division by their s.d.'s (calculations performed in Java). Cross-correlations of the *z*-score values were carried out in Java.

### ChIP-3C analysis

K562 or U2OS cells (10^7^ per sample) were fixed in 1% formaldehyde, quenched with 0.125 M glycine and then lysed with 0.2% Nonidet P-40 on ice for 1 h at 4 °C. Nuclei were collected by centrifugation, resuspended in NEB buffer 2 (New England Biolabs) containing 0.3% SDS, treated at 37 °C for 1 h and quenched with 2% Triton X-100 at 37 °C for 1 h, and then digested with HindIII at 37 °C overnight. The digested chromatin was immunoprecipitated with an antibody against RepID (A302-055A; Bethyl Laboratories Inc) or IgG control before proceeding to ligation. Subsequently, the beads were washed and resuspended in 200 μl of ligation buffer, and DNA was ligated at 16 °C overnight. Next, crosslinking was reversed and DNA was extracted by phenol/chloroform and ethanol precipitation. The interaction between HS2 and Rep-P was tested by PCR amplification (30 ng DNA template per reaction). The primers and probes used for PCR are listed in Supplementary Table 2. The 3C-qPCR assay primers were designed using the SnapGene Viewer Software. Real-time quantitative PCR was performed on ABI 7900 thermocycler using Taqman Premix (Invitrogen). Primer efficiencies were normalized using a single BAC (RP11622D14) clone covering the entire human ß-globin domain. 3C-qPCR data were normalized versus the ‘internal' primer pair AG (Supplementary Table 2) located in the *HBB* locus. The qPCR procedure (50 °C for 2 min followed by 95 °C for 10 min and 40 cycles of 95 °C for 15 s, 60 °C for 1 min) was performed according to the manufacturer's instructions.

### Chromosome conformation capture

Approximately 10^7^ cells were fixed in 1% formaldehyde, quenched with 0.125 M glycine and then lysed with 0.2% Nonidet P-40 on ice for 1 h at 4 °C. Nuclei were collected by centrifugation, resuspended in NEB buffer 2 (New England Biolabs) containing 0.3% SDS, treated at 37 °C for 1 h and quenched with 2% Triton X-100 at 37 °C for 1 h, and then digested with HindIII at 37 °C overnight. On the second day, DNA was ligated at 16 °C for 4 h in 7 ml of ligation buffer to minimize the intra-DNA ligation. Crosslinking was reversed and DNA extracted by phenol/chloroform. As a negative control, digested DNA was directly reverse-crosslinked without ligation. For the 3C assay, the HindIII-cut and re-ligated BAC RP11622D14 clone covering the entire human ß-globin domain was used for the 3C control templates. To correct for differences in digestion and crosslinking efficiencies between the different samples, 3C data were normalized towards loading controls (GAPDH primers) and a set of primers derived from the *ERCC3* gene^65^. Interaction among Rep-P origin and the human beta-globin locus was quantified with real-time PCR using an ABI 7900 thermocycler as described above (primers and probes used for real-time PCR are listed in Supplementary Table 2).

### Data availability

The data sets for the ChIP-seq and nascent-strand next-generation sequencing are available from the GEO under Accession codes GSE28911.

## Additional information

**Accession codes:** The data sets for the ChIP-seq and nascent-strand next-generation sequencing are available from the GEO under Accession codes GSE28911.

**How to cite this article:** Zhang, Y. *et al*. A replicator-specific binding protein essential for site-specific initiation of DNA replication in mammalian cells. *Nat. Commun.* 7:11748 doi: 10.1038/ncomms11748 (2016).

## Supplementary Material

Supplementary Information Supplementary Figures 1-7 and Supplementary Tables 1-4
