# Supplementary material for: A replicator-specific binding protein essential for site-specific initiation of DNA replication in mammalian cells
Source: Nat Commun. 2016 Jun 8;7:11748. doi: 10.1038/ncomms11748 (PMC4899857; doi:10.1038/ncomms11748)
Supplement: Supplementary Information — Supplementary Figures 1-7 and Supplementary Tables 1-4 [file ncomms11748-s1.pdf]

Supplemental Information:

**a**

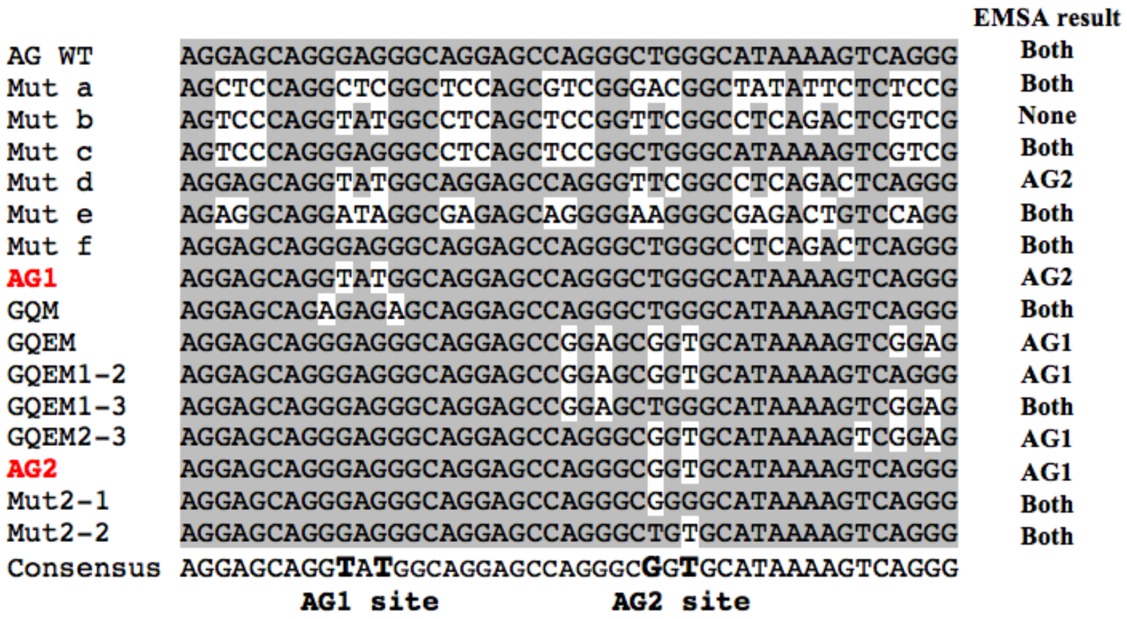


b

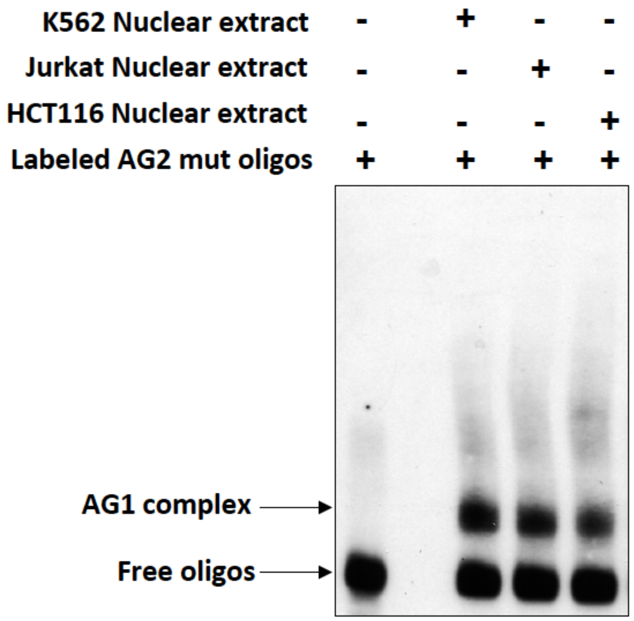

**Supplementary Figure 1| (a)** DNA sequence of all the oligonucleotides used in this study. Only one strand is shown. The unshaded nucleotide sequences show changes from the wild-type oligo (AG WT). The first 7 oligo mutants were designed to test AG asymmetry and to identify the AG1 binding site; the 8th to the 15th oligos were designed to identify the AG2 binding sites. A summary of the EMSA results using these oligos (for an example, see Fig. 1b) is listed on the right side panel. “None” indicates that no EMSA activities were observed; “Both” indicates that both AG1 and AG2 shifts were observed; “AG1” or “AG2” indicate the specific activity observed (see Fig. 1 of the main text for an example). (b) EMSA analysis using nuclear extracts from erythroid, non-erythroid cells and colorectal carcinoma cells with oligonucleotides containing the mutant asymmetric region (AG2, capable of forming the AG1 complex) from the Rep-P replicator.

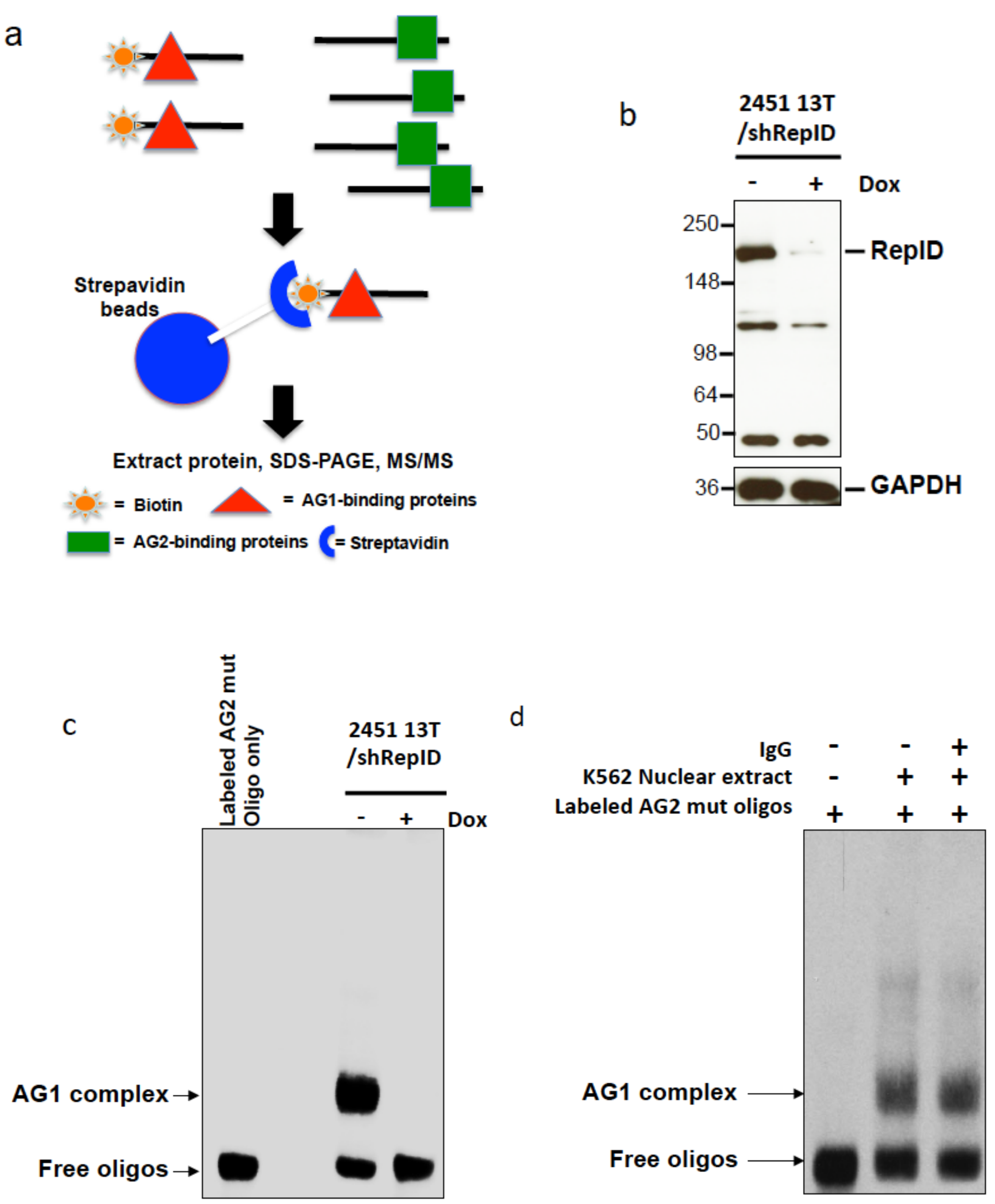
a
Strepavidin beads
Extract protein, SDS-PAGE, MS/MS
= Biotin
= AG1-binding proteins
= AG2-binding proteins
= Streptavidin
b
2451 13T /shRepID
- + Dox
250
148
98
64
50
36
RepID
GAPDH
c
Labeled AG2 mut Oligo only
2451 13T /shRepID
- + Dox
AG1 complex
Free oligos
d
IgG - - +
K562 Nuclear extract - + +
Labeled AG2 mut oligos + + +
AG1 complex
Free oligos

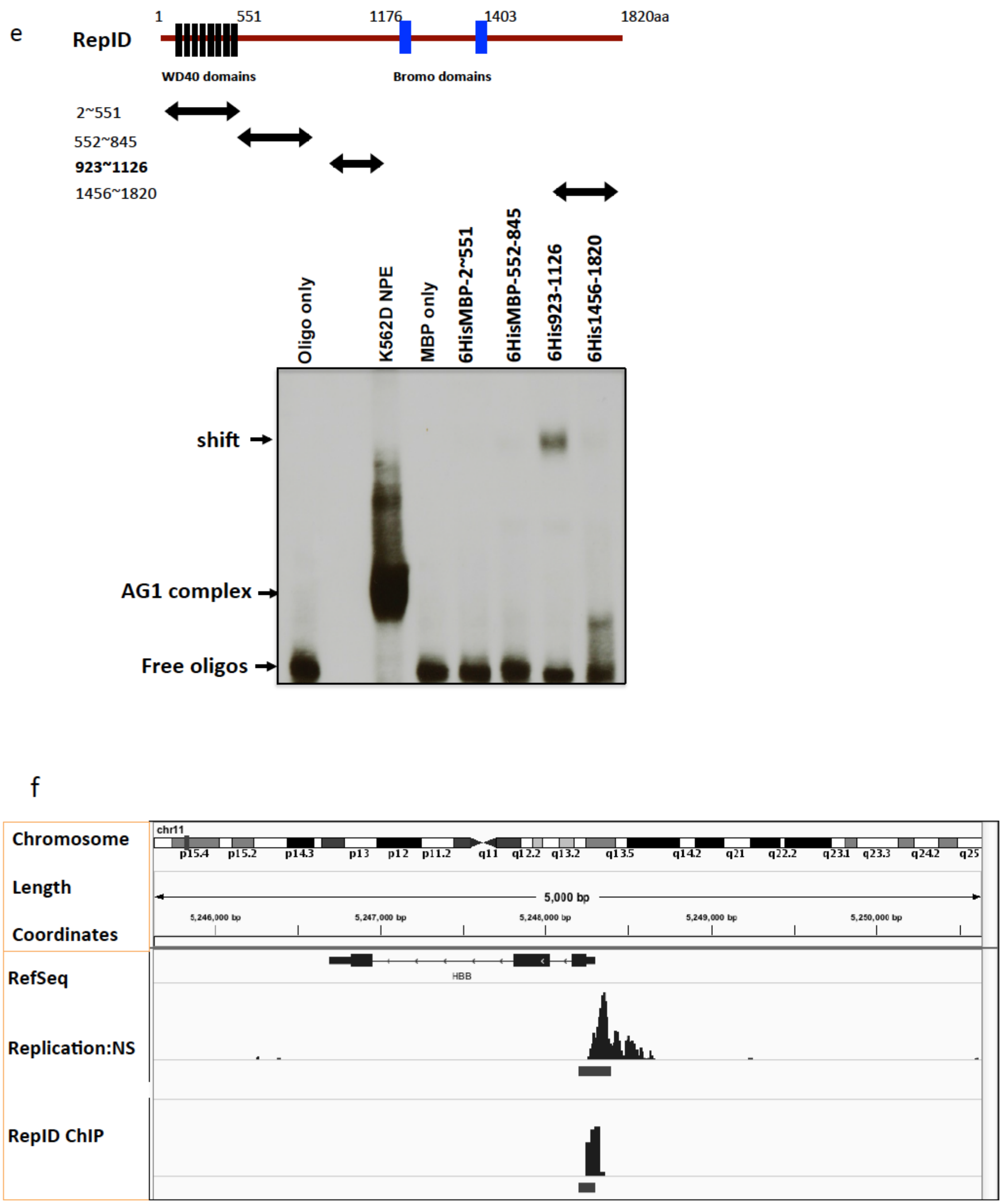


**Supplementary Figure 2**| RepID is an AG1 site-specific binding protein. (a) Identification of RepID. Principle of the biotin-pull-down assay used to isolate proteins that bind the AG oligo but not the mutant AG-1. Oligonucleotides with the AG1 site mutated were

added to the assay as a competitor to minimize non-specific binding and AG2-binding proteins. The resulting protein samples were sequenced by mass spectrometry (Tandem MS/peptide mapping). (b) 2451 13T melanoma cells stably expressing Tet-on inducible shRNA targeting RepID (2451 13T/shRepID) under the control of a doxycycline (Dox) promoter were treated with/without Dox for 16 days. Samples were analyzed by immunoblotting with a rabbit polyclonal antibody against RepID. The molecular weight of RepID is 206 kDa. Two additional non-specific bands were detected at 110kDa and 48kDa. (c) RepID depletion reduced AG1 binding activity sharply. EMSA was performed using nuclear extracts from melanoma 2451 13T cells stably expressing Tet-on inducible shRNA targeting RepID (2451 13T/shRepID), which were treated with/without Dox for 16 days, with oligonucleotides containing the AG2 mutation. (d) Prebleed (IgG) could not supershift a DNA-protein complex in EMSA with K562 nuclear proteins and oligonucleotides containing the AG2 mutation. (e) EMSA experiment was performed using from *E.coli* - purified RepID fragments and oligonucleotides containing the AG2 mutation to identify the DNA binding site within RepID. The illustration shows the full-length RepID protein, indicating the positions of the 8 WD40 domains and 2 Bromo domains and the positions of the purified fragments. (f) An IGV screenshot showing the replication initiation profile and RepID protein binding at the *HBB* locus obtained by massively parallel sequencing of nascent DNA strands (NS-Seq) and ChIP-Seq, respectively, in U2OS cells.

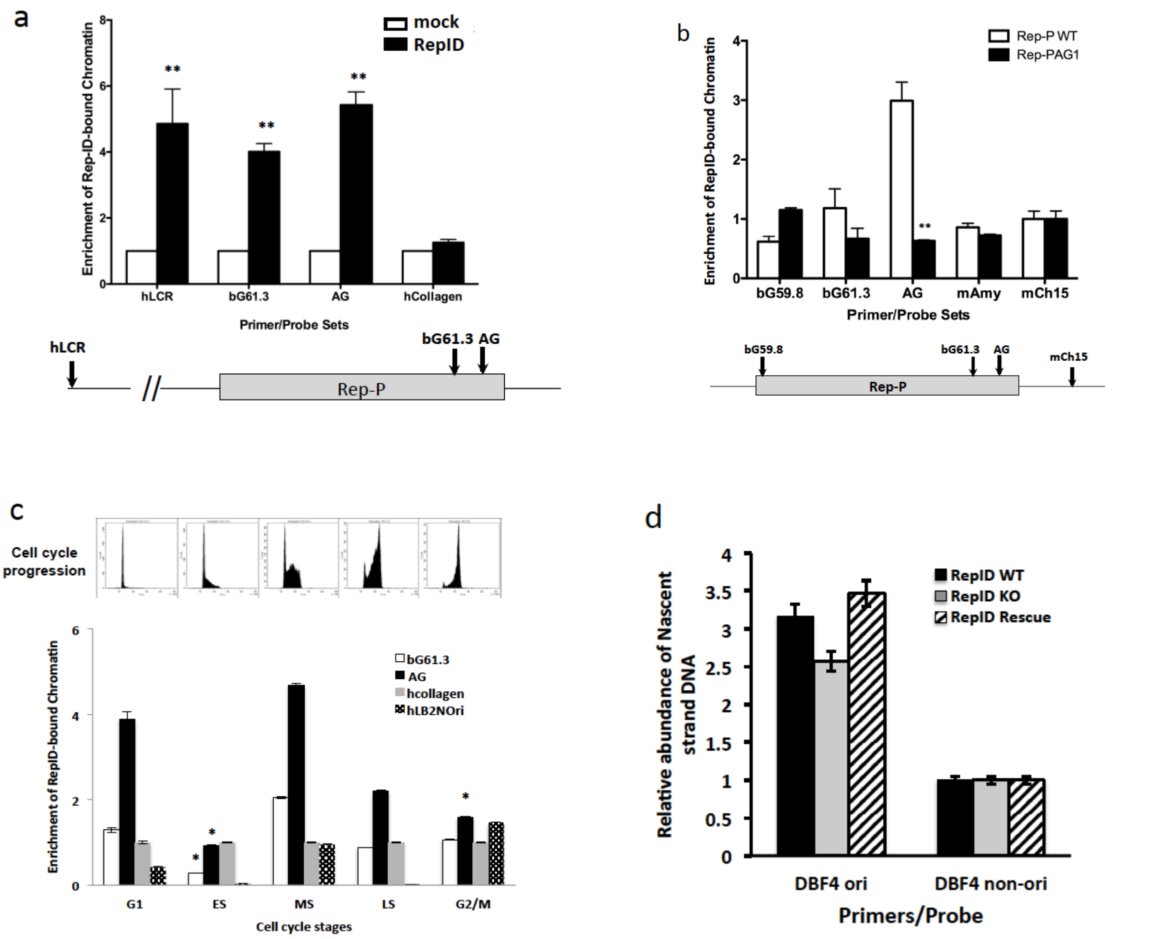


**Supplementary Figure 3**| Binding of RepID to sequences at the *HBB* locus. (a) ChIP identifies RepID binding at the LCR and at the Rep-P sequences within the *HBB l*ocus but not at a non-origin region (collagen) in K562 cells. The locations of primers from the *HBB* locus are shown underneath the histogram. This chart shows results from a representative experiment (n = 3). Statistical significance (P < 0.01**) was calculated using t-tests. (b) ChIP analysis of RepID binding in RL4 cells, derivatives from mouse erythroleukemia (MEL) that carry Rep-P WT or Rep-P AG1 mut transgene cassettes. For

RL4 cells, additional primers and probes used included mAmy (murine amylase), mCh15 (a murine sequence near the transgene insertion site). Q-PCR data were normalized as described in the "Methods" section and divided by the number of molecules amplified from the same preparation by the mCh15 primers. This chart shows results from a representative experiment (n = 3). Statistical significance ($P < 0.01$**) is indicated versus Rep-P WT as calculated using Student (t) tests. (c) ChIP analysis of RepID binding in lymphoma cells at different phases of the cell cycle. RepID was found at Rep-P replicator in G1 phase and middle S-phase. Primers and probes were used as listed in Fig. 2d. Q-PCR data are normalized as described in the "Methods" section. This chart shows results from a representative experiment (n = 3). Statistical significance ($P<0.05$*) was calculated versus G1 phase using t-tests. (d) The abundance of sequences from DBF4 origins and non-origins was measured in nascent (short, RNA-primed) DNA strands isolated from HCT116 cells harboring RepID sgRNA or rescued by expressing of pCMV-RepID-3×FLAG. Detailed experimental procedures were provided in the legend for Fig. 2f. Data are represented as the number of molecules amplified from RNA-primed nascent strands by the indicated primer divided by the number of molecules amplified from the non-origin primers. This chart shows results from a representative experiment (n = 3).

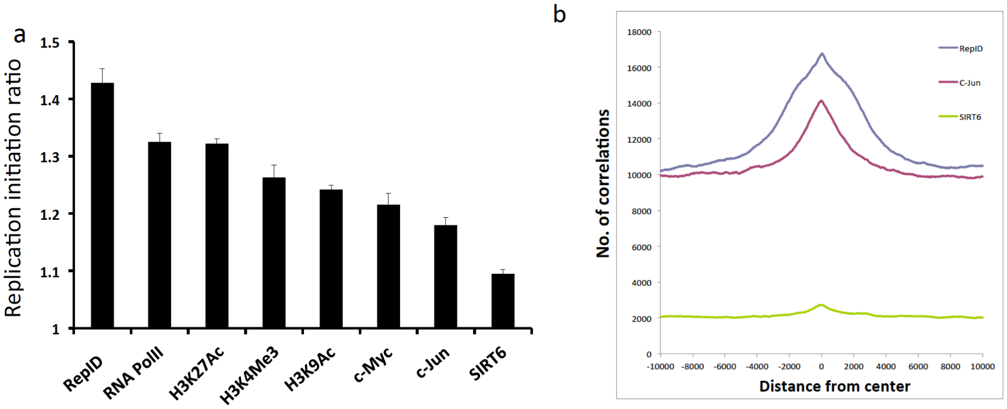


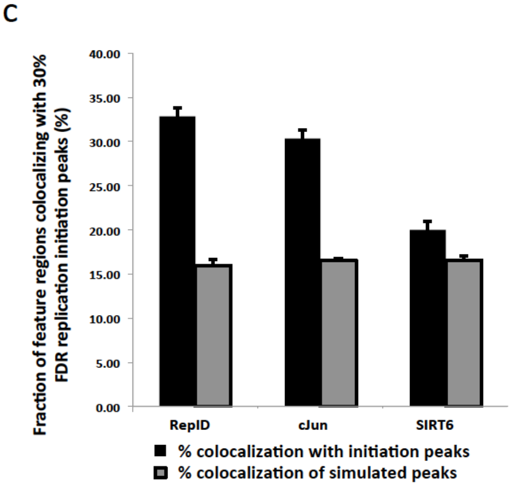


**Colocalization of RepID and other chromatin associated proteins with replication initiation peaks (FDR=0.15)**

| | RepID | cJun | SIRT6 |
|---|---|---|---|
| % colocalization with initiation peaks | 32.84 | 30.32 | 20.00 |
| % colocalization of simulated peaks | 15.96 | 16.55 | 16.56 |
| Standard deviation | 0.72 | 0.22 | 0.45 |

d

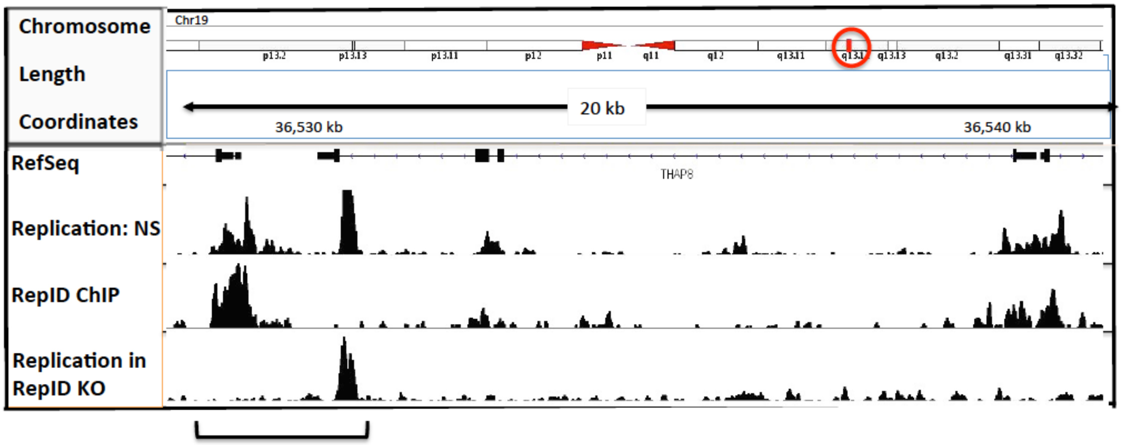
Chromosome
Length
Coordinates
Chr19
20 kb
36,530 kb
36,540 kb
RefSeq
THAP8
Replication: NS
RepID ChIP
Replication in RepID KO

e

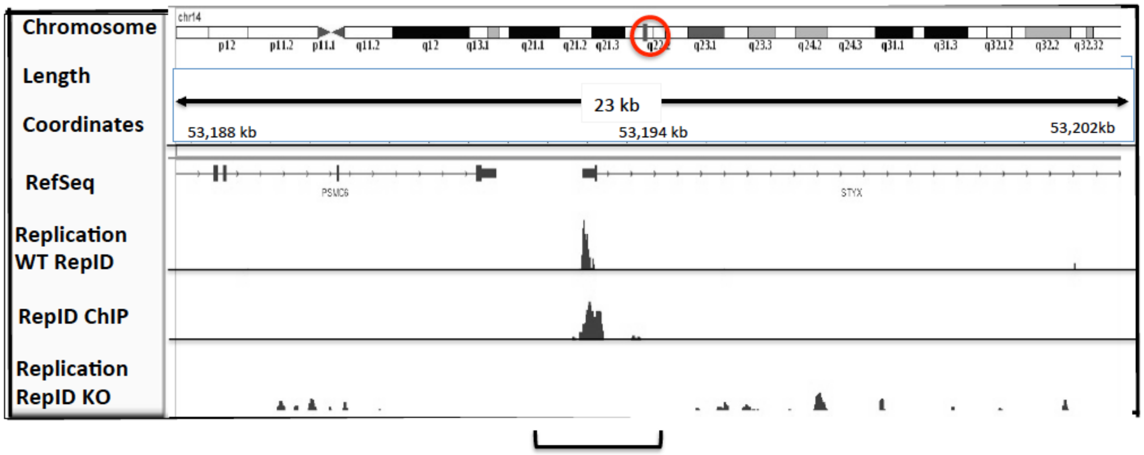
Chromosome
Length
Coordinates
chr14
23 kb
53,188 kb
53,194 kb
53,202kb
RefSeq
PSMC6
STYX
Replication WT RepID
RepID ChIP
Replication RepID KO

f

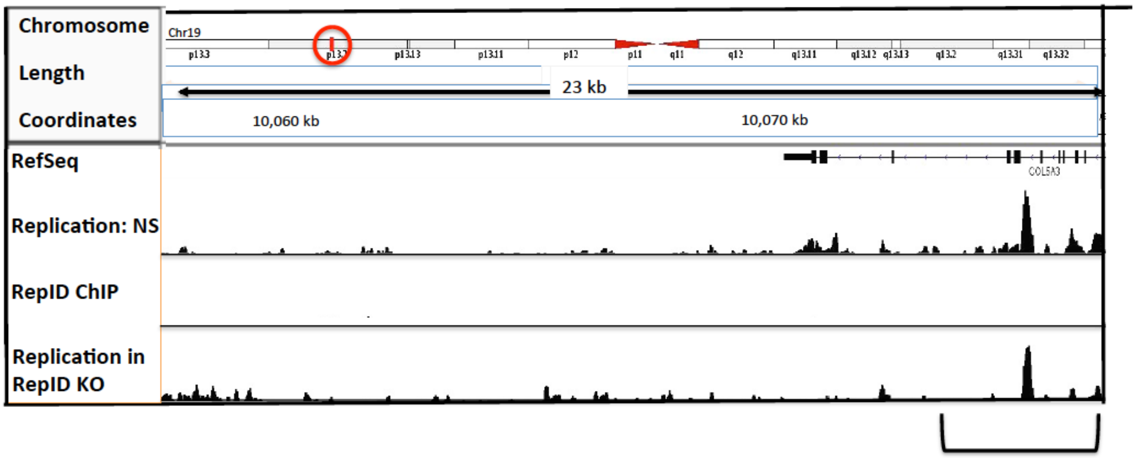
Chromosome
Length
Coordinates
Chr19
23 kb
10,060 kb
10,070 kb
RefSeq
COL5A3
Replication: NS
RepID ChIP
Replication in RepID KO

g

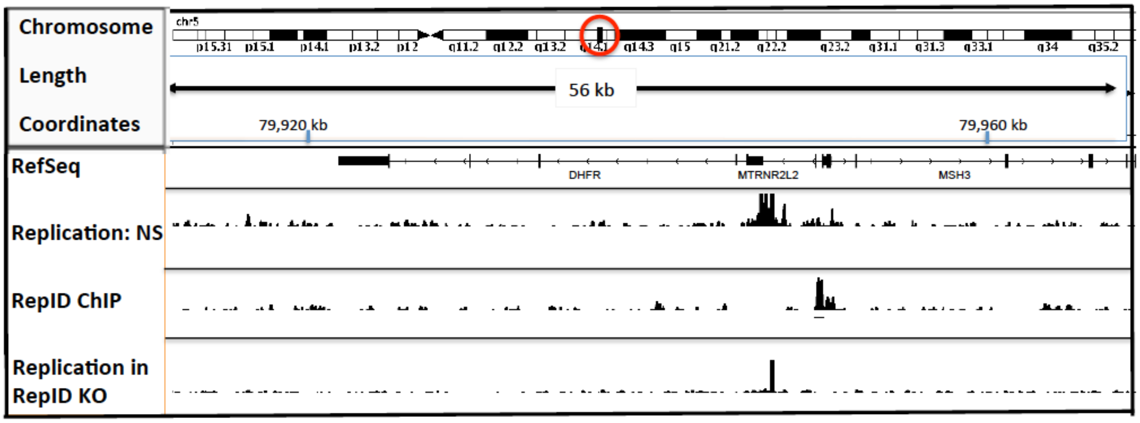
Chromosome
Length
Coordinates
chr5
56 kb
79,920 kb
79,960 kb
RefSeq
DHFR
MTRNR2L2
MSH3
Replication: NS
RepID ChIP
Replication in RepID KO

h

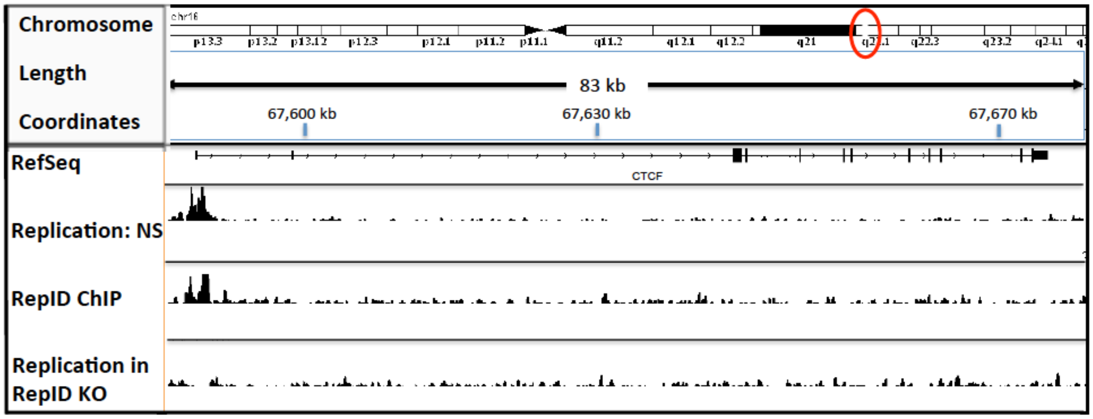


i

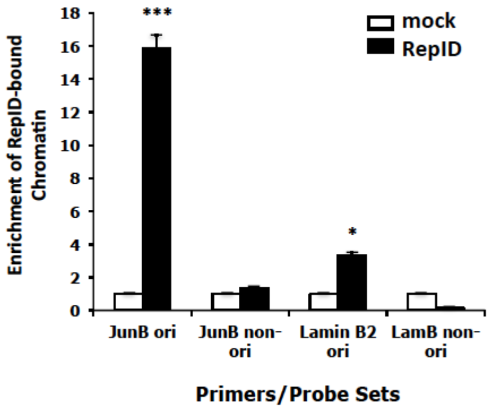


**Supplementary Figure 4|** Genome-wide colocalization of RepID with replication initiation sites. (a) Average genome-wide replication initiation enrichment ratios for protein binding sites in K562 cells. (b) Average genome-wide replication initiation ratios as a function of the distance from RepID, JunB and SIRT6 binding sites in K562 cells. JunB and SIRT6 binding site tracks were downloaded from the UCSC genome browser (*genome.ucsc.edu)* as described previously[5]. Replication initiation ratios were calculated from NS-Seq data as the ratios of reads obtained from a nascent strand preparation and reads obtained from the corresponding genomic DNA preparation as described [5]. Reads

were calculated as reads per kilobase per million of mapped reads (RPKM). (c) The fraction of RepID, c-Jun and SIRT6 binding sites colocalizing with replication initiation events. The fraction of colocalizing sites calculated from a theoretical distribution of randomly selected genomic regions of the same size (average of 100 simulations for each feature) is illustrated in comparison. This histogram represents the data shown on the right of the figure. (d) Screenshot of IGV browser tracks from the THAP8 locus on chromosome 19. The region of interest is marked on the ideogram with a red circle. RefSeq marks are shown below the chromosomal coordinates. The top track, immediately below the RepSeq genes, shows nascent strand patterns from cells with wild-type RepID (Replication: WT NS). The middle track shows ChIP-Seq patterns in WT cells (RepID ChIP). The lowest track shows nascent strand patterns from cells depleted of RepID (Replication: RepID KO NS). The underline bracket corresponds to the region shown in Figure 3a. (e) IGV screenshot of the STYX region on chromosome 14. Tracks layout is similar to (d). The underline bracket corresponds to the region shown in Figure 3d. (f) IGV screenshot of the COL5A3 region on chromosome 19. Tracks layout is similar to (d). The underline bracket corresponds to the region shown in Figure 3g. (g) IGV screenshot of the DHFR region on chromosome 5. Tracks layout is similar to (d). (h) IGV screenshot of the CTCF region on chromosome 16. Tracks layout is similar to (d). All the RepID-bound peaks shown in panels d through h, with the exception of the DHFR region in panel g, included the motif shown in Figure 3g.

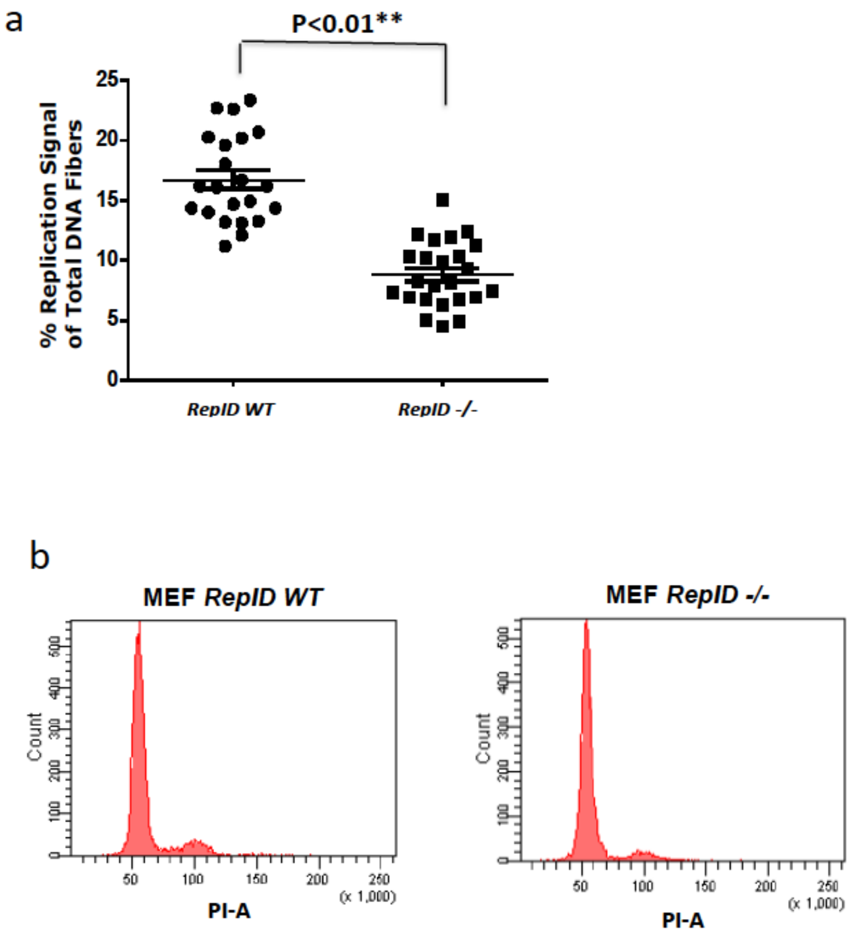
a
P<0.01**
% Replication Signal of Total DNA Fibers
25
20
15
10
5
0
RepID WT
RepID -/-
b
MEF RepID WT
MEF RepID -/-
Count
PI-A
50
100
150
200
250
(x 1,000)

C

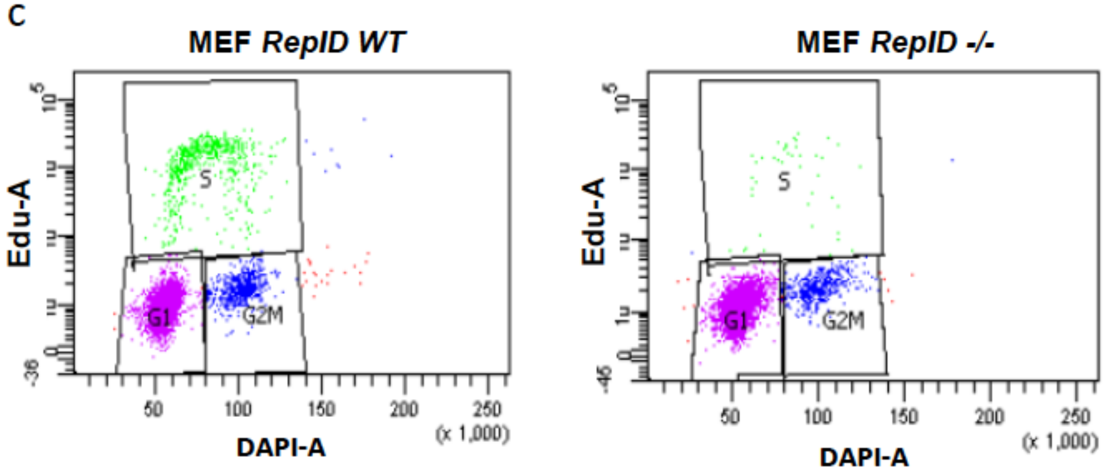


| | *RepID WT* | *RepID -/-* |
|---|---|---|
| **G1 Phase** | 82.6% | 91.7% |
| **S Phase** | 9.1% | 0.8% |
| **G2/M Phase** | 7.8% | 7.2% |

**Supplementary Figure 5** | Proliferation of MEFs that do not express RepID. (a) Frequency of DNA fibers that exhibit replication signals in wild-type and *RepID* depleted MEFs. The frequency was calculated as the percentage of fibers exhibiting replication signals (green or red signals >10 kb) vs. the total number of DNA fibers measured in both cells. Data from each cell type (WT and knockout) was taken with 8 random 6X13 montages. For each random montage, 13 fields were randomly selected to count the fibers. Approximately 500 fibers were measured to calculate the replication signals in every field. (b-c) Cell cycle profiles of *WT* and *RepID* depleted MEFs, and the statistic analysis data for EdU flow cytometry profiles.

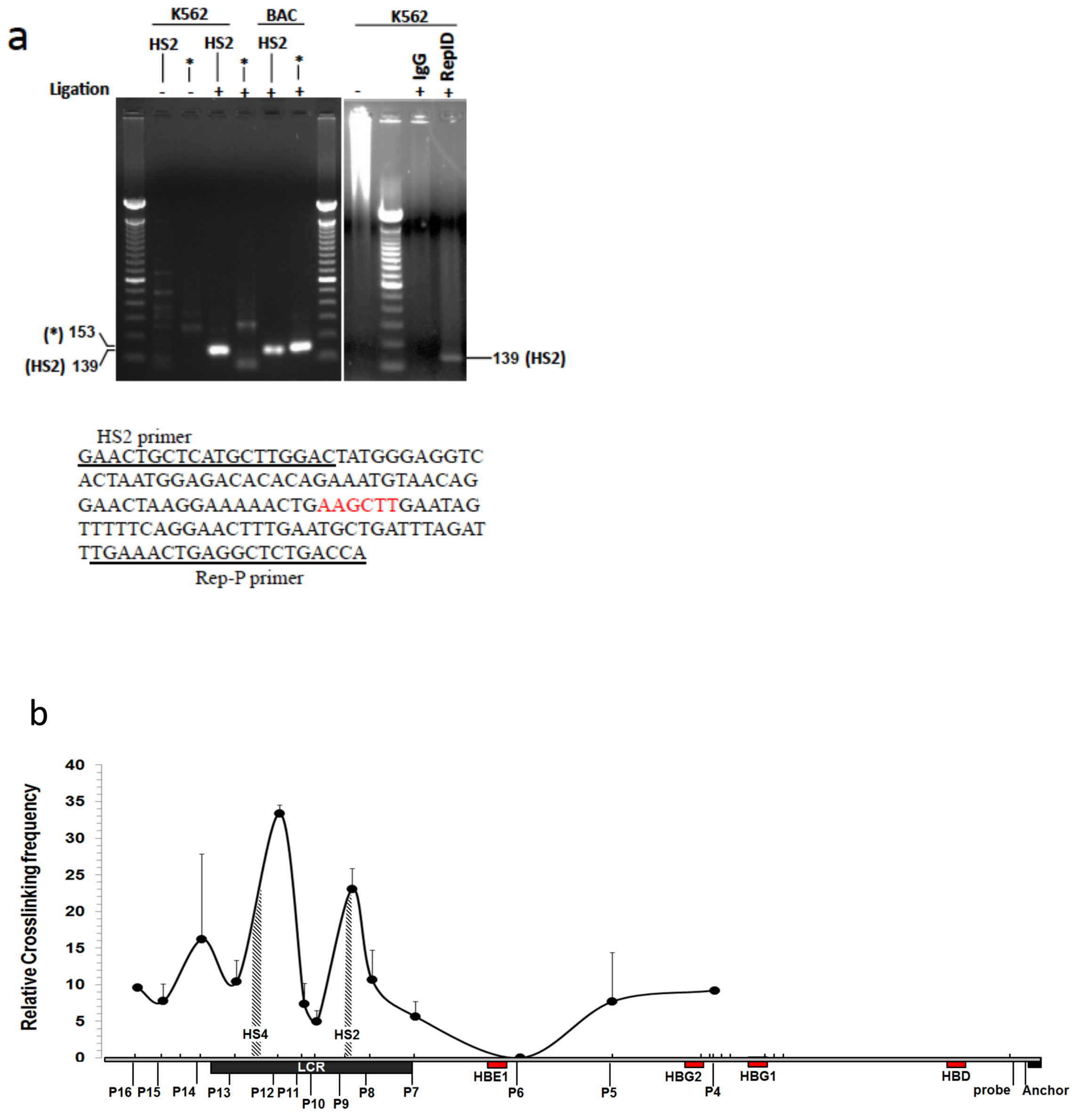


**Supplementary Figure 6**| RepID participates in a complex between LCR and Rep-P in early replicating *HBB* loci. (a) Nested PCR (left panel) and ChIP-3C (right panel) PCR analyzed by agarose gel electrophoresis. Only PCR2 is shown for nested PCR. Primers for a region showing low interaction by 3C qPCR (noted "*", and corresponding to primer 10 in S6b) did not show a PCR product in the nested PCR. A HindIII cut and religated BAC was used as a positive control (153 bp and 139 bp for "*" and HS2 respectively). Normal

IgG (IgG) or RepID (RepID) were added to the ChIP; the 3C procedure was performed with (+) or without the ligation step (-) as a negative control. *Bottom*, Sequence of the cloned PCR product corresponding to the 139 bp fragment. (b) HindIII-3C analysis of the long-distance interactions on the globin locus. The 3C primers correspond to sequences near the downstream sticky ends of the 3C fragments and were named p4 to p16 according to their position on the gene locus and are designated as half arrows. The x-axis shows the positions of the restriction fragments on the genomic scale (vertical bars). The data were normalized to the *ERCC3* locus. Error bars represent mean ± s.d for two independent experiments (n=2). Zones with diagonal lines denote the HS2 and HS4 core regions. See the Methods section for details.

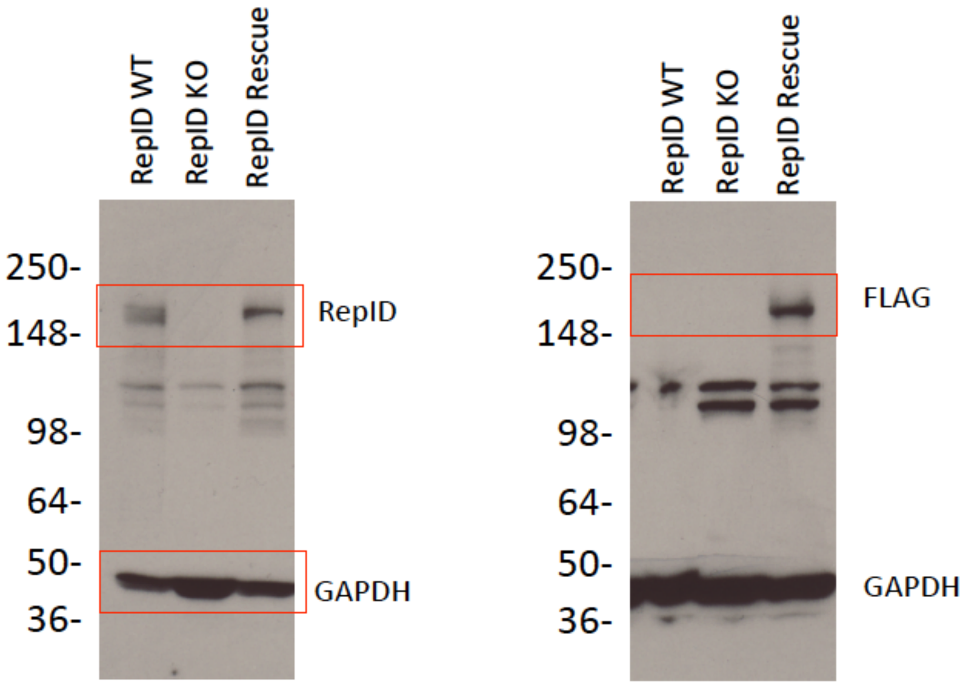


**Supplementary Figure 7. Original full blots for Fig. 2f.**

**Supplementary Table 1.** Transcriptional profiling of AG1-binding proteins suggests that RepID expression is coordinated with a group of proteins involved in DNA replication and repair

| | Pearsons correlation between transcript z scores | | | | | | | | | |
|---|---|---|---|---|---|---|---|---|---|---|
| | TNRC6A | ING4 | SMC2 | PARP1 | SND1 | C1orf156 | C9orf89 | RepID | BAZ2A | HMGB2 |
| TNRC6A | 1 | | | | | | | | | |
| ING4 | -0.11 | 1 | | | | | | | | |
| SMC2 | 0.17 | -0.05 | 1 | | | | | | | |
| PARP1 | -0.14 | 0 | 0.08 | 1 | | | | | | |
| SND1 | 0.05 | 0.1 | -0.13 | **0.31** | 1 | | | | | |
| C1orf156 | -0.11 | 0.09 | 0.22 | **0.32** | 0 | 1 | | | | |
| C9orf89 | -0.02 | 0 | 0.08 | **0.32** | **0.38** | 0.13 | 1 | | | |
| RepID | -0.04 | 0.17 | -0.06 | **0.31** | 0.04 | 0 | -0.13 | 1 | | |
| BAZ2A | -0.2 | 0.1 | **-0.34** | 0.1 | -0.2 | -0.05 | 0.01 | 0.05 | 1 | |
| HMGB2 | -0.17 | **0.29** | **0.41** | 0.17 | 0 | 0.18 | -0.1 | **0.39** | -0.16 | 1 |

This analysis was performed using the CellMiner tool (http://discover.nci.nih.gov/cellminer). CellMiner compares the expression patterns of groups of genes among the NCI-60 cancer cell lines and creates a Z-score matrix using the “z-score determination (include Cross-correlation)” option. Positive correlations were labeled with red bold fonts; negative correlations were labeled with blue bold font. The results showed significant correlations among several cell cycle associated genes and chromatin associated genes and RepID expression.

**Supplementary Table 2.** Primers and probes used in this study

| name | Forward primer | Reverse primers | Probe |
|---|---|---|---|
| **hLCR** | GGATCCACTTGCCCAGTGTT | TCTCAGCAGGGTTCAGGAAGA | TCCTTAGTTCCTACCTTCGACCTTGATCCTCCTT |
| **bG59.8** | TGGAAAAGCAACCCCTGC | AACTATGGATCCTTCTCTTGTGTTGG | GCTGCAGATACCATCATCCTGGCTTCAA |
| **bG61.3** | ACAGAGGCTTTTTGTTCCCCC | GGTAATCAGTGGTGTCAAATAGGAGG | GACACTCTTGCAGATTAGTCCAGGCAGA |
| **AG** | CAACTCCTAAGCCAGTGCCAGAAG | TGCCCTGACTTTTATGCCCAGC | TCATCACTTAGACCTCACCCTGT |
| **hCollagen** | TCTGAGAAGCCGTCCTCGTTA | CCAACAGTGGAGACACCCTTCTA | CGCCAGCCTGGCCTGTTCCA |
| **mAmy** | TCATATTCTAATCAAGACTAGTGACTTTAGAGC | TGCCACAACTACCAATCCTTTT | CAACTTCATTTCACACATGACTTTGCTGAGAAA |
| **mCh15** | TCCGTCCCCTTCTCCTCC | TTCAGGTTCCATTGCCACG | CACCATTCACACAGCCCACGAGCAAG |
| **DBF4ori** | GCCATGAGGATCCACAGTAA | CGAGGGGAGGAAAGGATTA | TCCTCCGCCTGCAGTCCCTT |
| **DBF4nonori** | AAGATTGTGCCACTGCACTC | TGAGGATGGGATGGACATAA | TCTCGCTCTGTTGCCCAGGTG |
| **JunBori** | GTGTATCCTGCGTCCGTGT | GCCTGCTGTCCTCTGTGA | |
| **JunBnonori** | CGACACAAGTTAGCCATAGGAA | CCCTGGATGCAAAGGTCTAT | |

## For 3C real-time PCR

| **Forward primer** | | |
|---|---|---|
| **Rep-P**: ACCAGGCAAATAAGTTTCAAGAA (anchor) | **Probe**: GAGGCTCTGACCATAACCAAATTTGCA | |
| **Reverse primers** | | |
| **P4**: GTTGACGCATGCCTAAAGAG | **P5**: GTTGTATATGTCCTATGGGATTTATGC | **P6**:CCAGATAGTACAGGGCCTGG |
| **P7**: CACAGATGCCTTAGCCTCAA | **P8**:TCAGCACAATGCCTAGGCTA | **P9**:TTCCAGCATCCTCATCTCTG (HS2) |
| **P10**: AACTGGGGACTCGAAAATCA | **P11**: GTCGAATGCAGCTGGTTAGA | **P12**:AATCTGTGAGCTCCTCACCAT (HS4) |
| **P13**: GGAACAGTTAAACAGCAACTACAGG | **P14**: TTGGCTCAAATGTCCTTGAAC | **P15**:CATGAGGAAACATTTTTTAAAGCC |
| **P16**:ATGAAGGCAGGTGACTCTAAC | | |
| **ERCC3 probe**: CAGTTGGGTGGGCTACACAGCAGTC | **ERCC3_1**: CCAGTTGTTAGGTTGGGAAAG | **ERCC3_2**: ACAGAAGCGGTGAGGTGAGTT |
| **GAPDH forward**: TACTAGCGGTTTTACGGGCG | **GAPDH reverse**: GAGGCTGCGGGCTCAATTT | |

**For nested PCR**

| | | |
|---|---|---|
| **Dpn1-Rep-P-3**: TGCTGCAGATACCATCATCC | **P17**: GTTTAGCATCCAGCAGGTGC | PCR1 (HS2) |
| **Rep-P**: ACCAGGCAAATAAGTTTCAAGAA | **P9**:TTCCAGCATCCTCATCTCTG (HS2) | PCR2 (HS2) |
| **Dpn1-Rep-P-3**: TGCTGCAGATACCATCATCC | **P18**:TCAGATGGTCTGAGCTCTCC | PCR1 (*) |
| **Rep-P**: ACCAGGCAAATAAGTTTCAAGAA | **P10**: AACTGGGGACTCGAAAATCA | PCR2 (*) |

**Supplementary Table 3. Cell lines used in this work.**

| Cell name | function |
|---|---|
| **MEF (Mouse Embryonic Fibroblast)** | Primary cells from RepID proficient and RepID deleted mice used to determine the effect of complete RepID depletion on cell cycle and DNA replication. |
| **CV-1** | Simian cells harboring either a Rep-P WT or Rep-P AG1 transgene cassettes in an identical genomic location. Cells were used to analyze the initiation of DNA replication in Rep-P mutants to neutralize chromosomal position effects and determine RepID binding patterns of WT and mutant Rep-P cells. |
| **RL4 MEL (Murine erythroleukemia)** | Similar to CV-1 cells, carried either a Rep-P WT or Rep-P AG1 transgene cassettes in an identical genomic location and orientation (RL4, "permissive" orientation). This cell was used to determine RepID binding patterns of WT and mutant Rep-P in erythroid cells. |
| **K562** | Human erythroleukemia cells that express the gamma globin in which the beta-globin locus replicates early during S phase. This cell line was used to determine RepID binding patterns at early replicating beta globin loci and to examine RepID chromatin binding throughout the cell cycle. |
| **353** | Human mantle lymphoma cells that replicate the beta-globin locus late during S phase. This cell line was used to determine RepID binding patterns at late replicating beta globin loci and to examine RepID chromatin binding throughout the cell cycle. |
| **HCT116** | Human colon cancer cells that replicate the beta globin locus late during the S-phase. These cells were chosen for further analyses because they express high levels of RepID as determined from the NCI-60 expression dataset. Cells were used in RepID interaction studies and to analyze the initiation of DNA replication in Rep-P origin following exposure to RepID directed single guide RNA. |
| **Jurkat** | Human T-cell lymphoma cells that replicate the beta globin locus late during S-phase. These cells were used to study RepID binding to the RepP region to investigate whether erythroid factors were required to facilitate that interaction. |
| **2451 13T (melanoma)** | Human patient melanoma cancer cells that expressed a high level of RepID proteins. Cells were used to validate antibody specificity following exposure to RepID directed shRNA and the effect of RepID depletion for AG1 complex formation. |
| **U2OS** | Human osteosarcoma cells that were used for depletion of RepID and for re-expression of FLAG-RepID in biochemical studies. Cells were used to RepID function in mediating interactions with distal sequences and regulation of initiation of DNA replication at the *HBB* locus. |

**Supplementary Table 4.** RepID motif localization

| | RepID Peaks | Randomized Peaks-1 | Randomized Peaks-2 | Randomized Peaks-3 |
|---|---|---|---|---|
| % with motif | 71.64 | 16.79 | 22.76 | 17.16 |
| % replication origins | 64.18 | 20.15 | 23.51 | 25.37 |
| % origins with motif | 70.35 | 31.48 | 42.86 | 30.88 |
| % non-origins with motif | 71.88 | 13.08 | 16.10 | 12.0 |

Percentages of RepID bound regions (or randomized files) that contain the motif (row 1), RepID bound regions that are replication origins (row 2), the percentage of RepID binding regions (or randomized files) that are also replication origins and contain the motif (row 3) and the percentage of RepID binding regions that are not origins and contain the motif (row 4).
